# A multi-domain lifestyle intervention in multiple sclerosis: a longitudinal observational study

**DOI:** 10.1007/s00415-025-13196-9

**Published:** 2025-06-24

**Authors:** Ilse M. Nauta, Keeva N. M. Loughlin, Arianne S. Gravesteijn, Jade van Wegen, Rosa P. Hofman, Nathalie Wilmsen, Emma Coles, Zoé L. E. van Kempen, Joep Killestein, Bob W. van Oosten, Eva M. M. Strijbis, Bernard M. J. Uitdehaag, Brigit A. de Jong

**Affiliations:** 1 Department of Neurology, MS Center Amsterdam, Vrije Universiteit Amsterdam, Amsterdam Neuroscience, Amsterdam UMC Location VUmc, P.O. Box 7057, 1007 MB Amsterdam, The Netherlands; 2 Division of Human Nutrition and Health, Wageningen University, Wageningen, The Netherlands; 3 MS Center Amsterdam, Rehabilitation Medicine, Vrije Universiteit Amsterdam, Amsterdam Neuroscience and Amsterdam Movement Science, Amsterdam UMC Location VUmc, Amsterdam, The Netherlands; 4 Voeding Leeft, Amsterdam, The Netherlands; 5 Quality of Care, Amsterdam Public Health Research Institute, Amsterdam, The Netherlands

**Keywords:** Health behavior, Mediterranean diet, Nutrition, Physical activity, Sleep, Relaxation

## Abstract

**Objective:**

To examine the effects of a multi-domain lifestyle intervention that advocated a Mediterranean style diet, and concurrently targeted physical activity, stress and sleep, on multiple sclerosis.

**Methods:**

A longitudinal observational study investigating the effect of a multi-domain lifestyle intervention (i.e., diet, exercise, stress, and sleep management) at four timepoints: start run-in, start and stop 3-month intensive interval, and 3-month follow-up. The primary outcome (i.e., impact of multiple sclerosis on daily functioning) and secondary outcomes (i.e., quality of life, general health, multiple sclerosis-specific symptoms, and lifestyle factors) were analyzed using mixed models. Analyses were repeated among subgroups based on program compliance, body mass index, education level, and multiple sclerosis-subtype.

**Results:**

Out of 668 participants, 579 were included (age 46.2 ± 10.5 years, 84.5% women, and 71% relapsing–remitting multiple sclerosis). The impact of multiple sclerosis on physical functioning remained stable during the run-in period, but reduced significantly from baseline to both post-intervention (*β* = −2.50 [−3.40, −1.60]) and to 3-month follow-up (*β* = −2.00 [−2.93, −1.07]). The impact of multiple sclerosis on mental functioning decreased significantly across all time periods (run-in *β* = 1.86 [0.78, 2.94], post-intervention *β* = −3.48 [−4.58, −2.39], and 3-month follow-up *β* = −2.44 [−3.56, 1.31]). Effect size was greatest among participants with higher compliance, lower education, and obesity.

**Interpretation:**

The lifestyle program was associated with reduced impact of multiple sclerosis on daily functioning, multiple sclerosis-related symptoms, mental quality of life, and general health determinants. Future randomized trials are needed to establish causal effects of lifestyle adjustments on multiple sclerosis.

**Supplementary Information:**

The online version contains supplementary material available at 10.1007/s00415-025-13196-9.

## Introduction

Multiple sclerosis (MS) is a chronic inflammatory, demyelinating, neurodegenerative disease affecting the CNS. MS likely arises from an interaction between genetic predisposition, lifestyle, and environmental risk factors [1, 2]. Although there is no cure for MS, numerous immune-modulating, disease-modifying therapies (DMTs) are available. While these DMTs are effective in suppressing inflammatory disease activity, their impact on slowing disease progression is less evident and may be accompanied with side-effects [2]. This, but also the urgent desire of the people with MS to regain control over their disease adds to a growing interest in additional disease management strategies aimed at alleviating MS symptoms and preventing disease progression [1, 3].

Modifiable lifestyle factors, particularly dietary intake, have gained increased attention in the management of MS symptoms. Although robust, large-scale longitudinal trials are still lacking [4, 5], multiple studies have associated adherence to a healthier diet (e.g., higher intake of vegetables, fruit and whole-grains; lower intake of sugar and red meat) with fewer MS symptoms, including lower physical disability, reduced fatigue, less depressive symptoms, and better overall quality of life [6–8]. The exact mechanism(s) how a healthier diet and other lifestyle factors are associated with better MS outcomes remains unknown. Several factors may apply, including the positive effect of reducing comorbidities, reduced physical disability enabling easier access to a healthier lifestyle, and the anti-inflammatory and neuromodulating properties of a healthier diet [6, 8, 9]. Adherence to a more pro-inflammatory diet is associated with worse clinical and MRI characteristics [10], whereas an enhanced anti-inflammatory diet, such as the Mediterranean diet (MD) [11], has been linked to less physical disability, lower fatigue levels, higher quality of life (QoL), and better imaging outcomes in MS [8, 12–14].

Along with diet, other modifiable lifestyle factors, such as physical activity, stress management, and sleep quality, seem promising targets in the management of MS symptoms. Physical activity may reduce MS-related fatigue, pain, immobility, cognitive complaints [15], and moderate-to-vigorous exercise may ameliorate the neuroinflammatory [16] and neurodegenerative aspects [17] of the disease [18]. Over the years, consistent data show an association between stressful life events and an increased risk of exacerbations of MS [19]. Adequate stress management among MS patients may reduce perceived stress and improve QoL [20]. Decreased sleep quality is frequently reported among MS patients and is associated with other MS-related symptoms, including pain, fatigue, mood disorders, and cognitive dysfunction [21]. Even though individual lifestyle factors show positive associations with fewer MS symptoms, studies on multi-modal lifestyle interventions in MS, where multiple factors are targeted simultaneously, are scarce [22, 23]. The effects of multi-domain lifestyle adaptations on MS symptoms should consequently be studied in large-scale longitudinal studies among MS patients.

Here, we performed a longitudinal, observational study among a large cohort of participants with MS to investigate the effect of multifaceted lifestyle adaptations on impact of MS on daily functioning. Additionally, we investigated QoL, general health, MS-specific symptoms, and lifestyle factors. The online multi-domain lifestyle intervention advocated an adapted MD, while concurrently targeting physical activity, stress, and sleep.

## Methods

### Study design

**Fig. 1:**
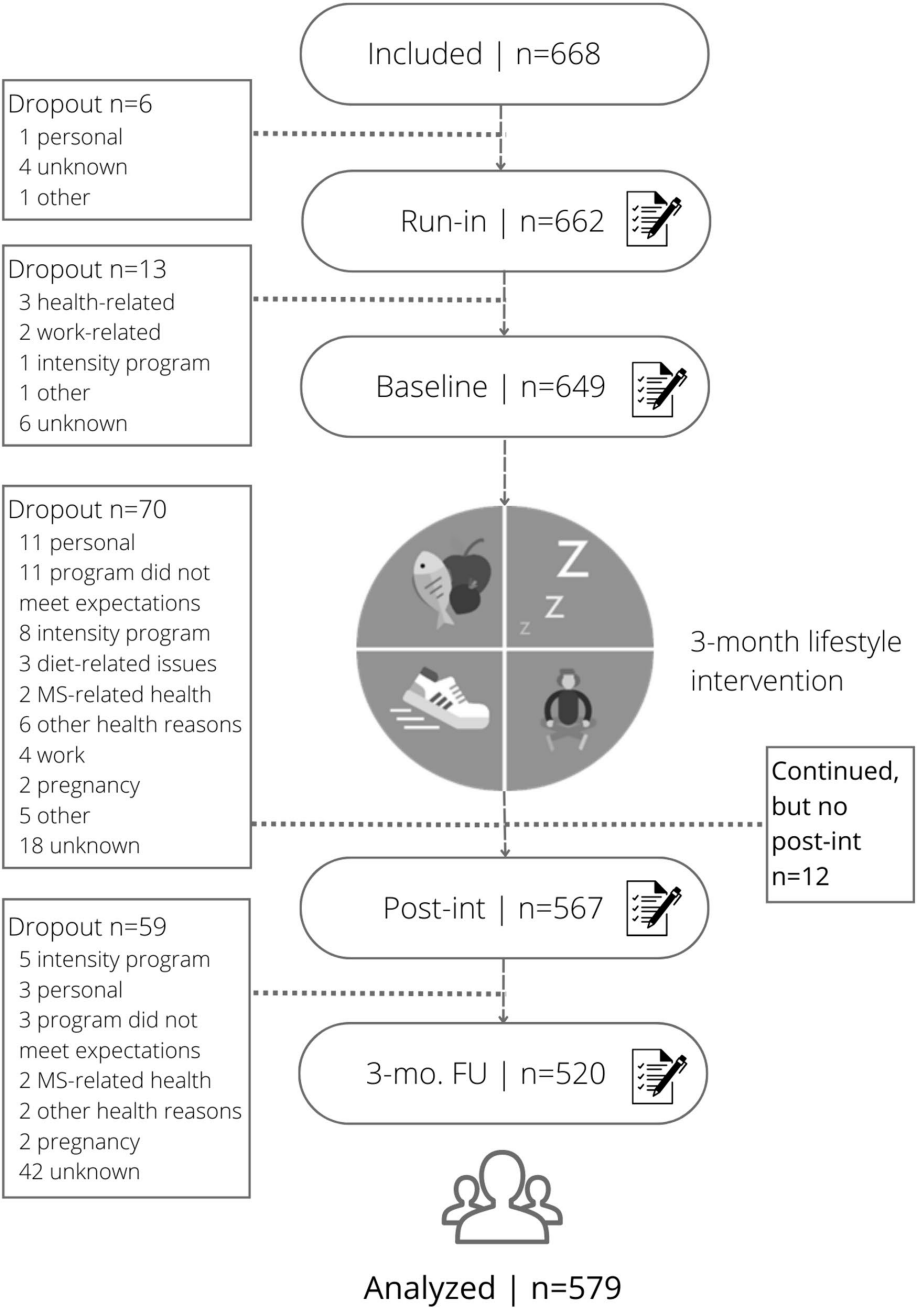
Flowchart of the LIMS study. Abbreviations: Post-int = post-intervention measurement; 3-mo. FU = 3-month follow-up measurement

The ‘Lifestyle Intervention in MS’ (LIMS) study is a longitudinal and observational study where patients with MS participated in a 3-month intensive online 3-month multi-domain lifestyle program followed by a 21-month after-care period, called “Live! with MS” (*in Dutch: ‘Leef! met MS’*). This program was developed and delivered by the Dutch Foundation “Nutrition Lives” (*in Dutch ‘Voeding Leeft’*). Participants served as their own control during a 3-month run-in period prior to the start of the intervention. During the run-in period, no recommendations on lifestyle changes or other recommendations were provided. This manuscript covers four measurement points of the program (Fig. 1): 3 months prior to the intervention (run-in), the start of the 3-month intensive program (baseline), directly following intervention completion (post-intervention), and 3 months following intervention completion (3-month follow-up). The complete follow-up duration of the LIMS study is 21 months and is still ongoing. All assessments consist of online patient-reported outcome measures (PROMs). The PROMs for current manuscript were collected between June 2021 and June 2023. Eight groups (40–100 participants per group) completed the run-in measurement between June 2021 and August 2022, and subsequently started the lifestyle program. Medical ethical approval was obtained from the institutional ethics review board of the Amsterdam UMC (2021.0089). The procedures in this study comply with the ethical standards of the relevant national and institutional committees on human experimentation and with the Helsinki Declaration of 1975, as revised in 2008. Online informed consent was obtained from all participants. The study was registered at ClinicalTrials.gov (Identifier: NCT05402501; https://classic.clinicaltrials.gov/ct2/show/NCT05402501; Study Start 2021-04-15).

### Participants

Recruitment took place via websites and social media of the National MS Foundation, MS Center Amsterdam, and the Foundation Nutrition Lives, all based in the Netherlands. MS patients interested in participating in the study could apply through an online form. The Foundation Nutrition Lives monitored and registered participants and checked their eligibility. Participants were eligible if they had a confirmed diagnosis of MS according to the McDonald 2017 criteria [24], were aged 18–75 years at the start of the lifestyle program, were motivated to adapt their lifestyle, could independently complete the online PROMs, and were able to do grocery shopping and prepare the meals from the program (either by themselves or with help from others). From each potential participant, the diagnosis of MS was checked by requesting the medical letters of the treating neurologists and the MRIs, if this information could not be provided or the received information did not support the diagnosis of MS, the patient was excluded from participation. In addition, participants were excluded if any of the following criteria applied: unable to participate in an online lifestyle program (e.g., not in possession of an e-mail address, laptop/computer, and Internet), unable to speak or read Dutch, consuming a vegan diet, unwillingness to eat fish, Body Mass Index (BMI) < 18.5 or > 35 kg/m^2^, pregnant or lactating, diagnosed with an eating disorder, psychiatric disorder, inflammatory bowel disease (e.g., Crohn’s disease or ulcerative colitis), diabetes type 1 or 2, malignancies or moderate-to-severe cardiovascular disease, a history of bariatric surgery, previous or current participation in a lifestyle program, or currently being treated by a dietician or lifestyle coach.

### Lifestyle program

The lifestyle intervention “Live! with MS” of Foundation Nutrition Lives (based on their currently reimbursed program “Reverse Diabetes2 Now” for patients with diabetes mellitus type 2 in the Netherlands lifestyle[25]) had a total duration of 2 years, which consisted of a 3-month intensive period and a 21-month after-care period. The program addressed four lifestyle domains: diet, physical activity, relaxation, and sleep. The intervention and information during the program were provided in a standardized manner using pre-defined protocols.

During the 3-month intensive period, three main meetings (5 h; groups of ± 100 patients, split into groups of 25 patients with a coach per sub-group) and five theme hours (1 h; fixed groups < 25 patients) provided information on healthy lifestyle strategies to implement in daily life (for content of all the meetings, see Supplementary Table 1). In addition to these meetings, participants had access to an online community where participants could share their experiences, ask questions and participate in an online community and e-learning environment during the whole program. In addition, dieticians and lifestyle coaches provided guidance during the meetings and within the online environment. The focus of the lifestyle intervention was *diet*. Participants received instructions on how to adopt a modified MD, in line with the Dutch dietary guidelines (The Dietary Guidelines 2015 of the Health Council of the Netherlands). The dietary advice emphasized consuming unprocessed foods, increasing vegetable intake, consuming whole-grain products, having three satisfying meals per day without snacks, and abstaining from alcohol consumption. The specific dietary recommendations are provided in Supplementary Table 2. Participants received recipes for three meals per day, during a period of 4 weeks. *Physical activity* was addressed during the second month of the lifestyle program with the goal of increasing participants’ physical activity levels [26]. Participants set out feasible, individual physical activity goals. The goals sought to cover three basic principles: (1) exercise 15-to-30 min a day with increased heart rate, (2) good distribution of movement throughout the day, and (3) the activities should be suitable and achievable. Based on these goals, participants followed a physical activity plan tailored to their baseline activity level. *Relaxation* was covered during the third month of the program. Participants were provided with advice and exercises, requiring minimal effort, to enhance recognition of stress responses, identify personal approaches for daily relaxation, and to breathe more consciously. The last main meeting was used to inform and advise participants on how to improve *sleep* and develop a personal sleep routine. Throughout the 3-month program, coaching on behavioral change was also provided. Overarching themes were addressed including increased acceptance of their MS diagnosis, coping with physical limitations, and rebuilding self-efficacy.

After the intensive period, participants followed a 21-month after-care program. During this period, meetings were offered less frequently, focused on refreshing knowledge provided in the first three months and coaching patients to adhere to the program for the longer term. Patients retained access to the online environment. In the current study, we included the first 3 months of this after-care program. During this 3-month after-care period, a refresher session on nutrition and a coaching session were provided. More detailed information on the content of the lifestyle program can be found in Supplementary Table 2 and Supplementary Methods.

### Outcomes

At the start of the run-in period, information on demographics, clinical background, and comorbidities were collected. At the completion of the intensive period, meeting attendance was assessed and participants were asked to evaluate the lifestyle program. All outcomes were assessed through online questionnaires.

At all four assessment moments, impact of MS on daily functioning, QoL, general health, MS-specific symptoms, and lifestyle factors were administrated. The primary outcome was the change of *impact of MS on daily functioning*, both the physical and psychological component, measured with the Multiple Sclerosis Impact Score (MSIS-29, comprises 20 items on physical impact and 9 items on psychological impact) [27]. Scores for each scale were converted into 0–100 scores, and lower scores indicated a lower impact of MS. In addition, the number of participants who reached the minimally clinically important difference (MCID) on the MSIS physical impact scale were determined. Costelloe et al. [28] showed that a change of 8.0 points is considered the MCID for MSIS physical impact, the MCID for the psychological impact of the MSIS-29 is yet to be determined. *QoL* was measured with the Short-Form health survey (SF-12) [29], which includes both physical and mental health-related QoL. *General health* included BMI (kg/m^2^), waist circumference (cm), and classifying the form of feces according to the Bristol Stool Scale [30]. For BMI and waist circumference, participants measured their weight and waist with their own scale and tapeline, respectively. Participants were asked to list—if applicable—their comorbidities beside their diagnosis of MS. *MS-specific symptoms* including depression and anxiety were measured using the Hospital Anxiety and Depression Scale (HADS) [31], fatigue using the Checklist Individual Strength-20-r (CIS-20)—subjective tiredness[32] and patient-reported cognitive complaints using the Multiple Sclerosis Neuropsychological Screening Questionnaire—patient version (MSNQ-p) [33]. Lifestyle outcomes consisted of adherence to the MD (checklist based on the assessment tool of Martinez-Gonzalez et al. [34], maximum score of 17), adherence to the Dutch exercise guidelines[26] (using the subscales moderate-intensive, muscle-bone, and total exercise) [35], stress (perceived stress scale (PSS)) [36], and sleep quality (Medical Outcomes Study Sleep Scale (MOS-ss; 9 subscales)) [37].

### Statistical analysis

Data were analyzed in SPSS 26 and STATA 17. Anxiety and depression data were log-transformed. MOS-ss subscales snoring and short of breath were dichotomized (median split). Linear, binary and ordinal mixed model analyses were performed for normally distributed, binary and ordinal outcomes, respectively. Participants were included as random factors and time (i.e., run-in, baseline, post-intervention, 3-month follow-up; baseline coded as reference) as a fixed factor. The change over time in the run-in period was compared with the change after completion of the intensive period of the lifestyle intervention. A modified intention-to-treat approach was used, including all participants with at least one follow-up measurement regardless of their compliance to the program. Age and sex were inserted as covariates.

These mixed model analyses were repeated among highly compliant participants, and in normal, overweight, and obese participants separately. High compliance was defined in three ways: (1) high attendance, defined as attending all main meetings and > 50% of the theme hours, (2) high attendance *and* the upper tertile of MD change between baseline and post-intervention, and (3) high attendance *and* the upper tertile of MD adherence at post-intervention.

To investigate the generalizability of the intervention effects, the primary outcome (MSIS-29) was also analyzed in the following ways: (1) in the main sample, with age, sex, disease duration, DMT use, MS type, and BMI as covariates; (2) separately in participants with relapsing and progressive MS, and (3) separately in participants with lower and higher education levels. Finally, the percentage of participants who reached an MCID of the physical MSIS-29 during at least one follow-up assessment was determined.

Alpha was set at 0.05 (two-tailed), and Bonferroni-corrected if multiple subscales were analyzed (0.05/number of scales).

## Results

### Participants

Out of the 668 MS patients enrolled, 579 participants (mean age 46.2 ± 10.5 years, median BMI of 25.6 kg/m^2^, 31% overweight, 18% obese, 84.5% women, and 71% relapsing–remitting MS) had at least one follow-up measurement and were included in the analyses. See also Table 1. The percentage of enrolled women was somewhat higher as in general reports on the female-to-male ratio among patients with MS [38]. The presence of participants with overweight was similar to the general Dutch population (https://www.rivm.nl/en/lifestyle-monitor). Figure 1 presents the flowchart of the study and Table 1 shows the participants’ characteristics at the first measurement (run-in). Participants who dropped out during the program (between run-in and post-intervention; *n* = 83) differed from the analyzed participants (*n* = 579) regarding smoking (*P* =.021): 56% had never smoked in the analyzed sample compared to 45% in the drop-out sample, whereas the analyzed sample had a lower percentage of patients who had stopped smoking (41%) or still smoked (3%) compared to the drop-out sample (47% and 8%, respectively), which may indicate that the participants who remained in the program had in general a healthier lifestyle when compared to the drop-outs. No other demographic, disease-related, or general health characteristics differed between the groups (*P* >.05).

**Table 1:** Baseline characteristics of the study population

Baseline characteristics	Participants (*n* = 579)
*Demographics*	
Age (years), mean (SD)	46.2 (10.5)
Sex (women), *n* (%)	489 (84%)
Education (low/middle/high),* n* (%)	4/28/68%
*Disease-related characteristics*	
MS subtype (RRMS/SPMS/PPMS/subtype not defined), %	71/9/12/8%
Symptom duration (years), median (IQR)	10.8 (14.1)
Diagnosis duration (years), median (IQR)	7.2 (11.5)
DMT (yes), *n* (%)	374 (65%)
*Self-reported general health*	
BMI (kg/m^2^), mean (SD)^a^	25.6 (4.5)
Underweight, *n* (%)	5 (1%)
Normal, *n* (%)	288 (50%)
Overweight, *n* (%)	174 (31%)
Obese, *n* (%)	104 (18%)
Waist (cm), mean (SD)^b^	92.0 (13.3)
Low risk, *n* (%)	124 (21%)
High risk, *n* (%)	161 (28%)
Very high risk, *n* (%)	290 (50%)
Smoking (yes, quit, never), %	3/41/56%
Alcohol (never, ≤ 2 days/wk, ≥ 3 days/wk), %	36/52/12%
Vegetarian (yes), *n* (%)	49 (8%)
Vitamin D	326 (56%)
Vitamin C	132 (23%)
Vitamin B12	110 (19%)
Magnesium	140 (24%)
Omega-3	97 (17%)
Self-reported comorbidities (yes), *n* (%)^c^	175 (30%)
Cardiovascular disorder (mild)	56 (10%)
Allergy	39 (7%)
Psychological disorder	34 (6%)
Skin disease	23 (4%)
Muscle and/or bone comorbidities	16 (3%)
Thyroid disorder	16 (3%)
Gastrointestinal disease	13 (2%)
Other neurological disease	12 (2%)
Migraine	8 (1%)
Rheumatic condition	8 (1%)
Sleep disorder	6 (1%)
Other	54 (9%)

^a^BMI: underweight < 18.5, normal 18.5–24.9, overweight 25.0–29.9, Obese > 30

^b^Women: low risk < 80 cm, high risk 80–88 cm, very high risk > 88 cm; Men: low risk < 94 cm, high risk 94–102 cm, very high risk > 102 cm

^c^The individual supplement types and comorbidities do not add up to 100%, as participants could report multiple supplements or multiple comorbidities simultaneously, or none at all

*SD*  standard deviation, *RRMS* relapsing–remitting multiple sclerosis, *SPMS* secondary progressive multiple sclerosis, *PPMS* primary progressive multiple sclerosis, *IQR* interquartile range; *DMT* disease-modifying therapy; *BMI* body mass index

### Attendance

Out of the participants who completed the post-intervention measurement (*n* = 567), 69% attended all three program days, 24% attended two, and 6% attended one program day. 520 participants also completed the 3-month follow-up measurement of which 51% attended the refresher coaching session and 57% attended the refresher webinar on nutrition.

### Intervention effects

Supplementary Table 3 presents the outcome values at each time-point, and Supplementary Table 4 shows the changes over time.

#### Primary outcomes: impact of MS on daily functioning

**Fig. 2:**
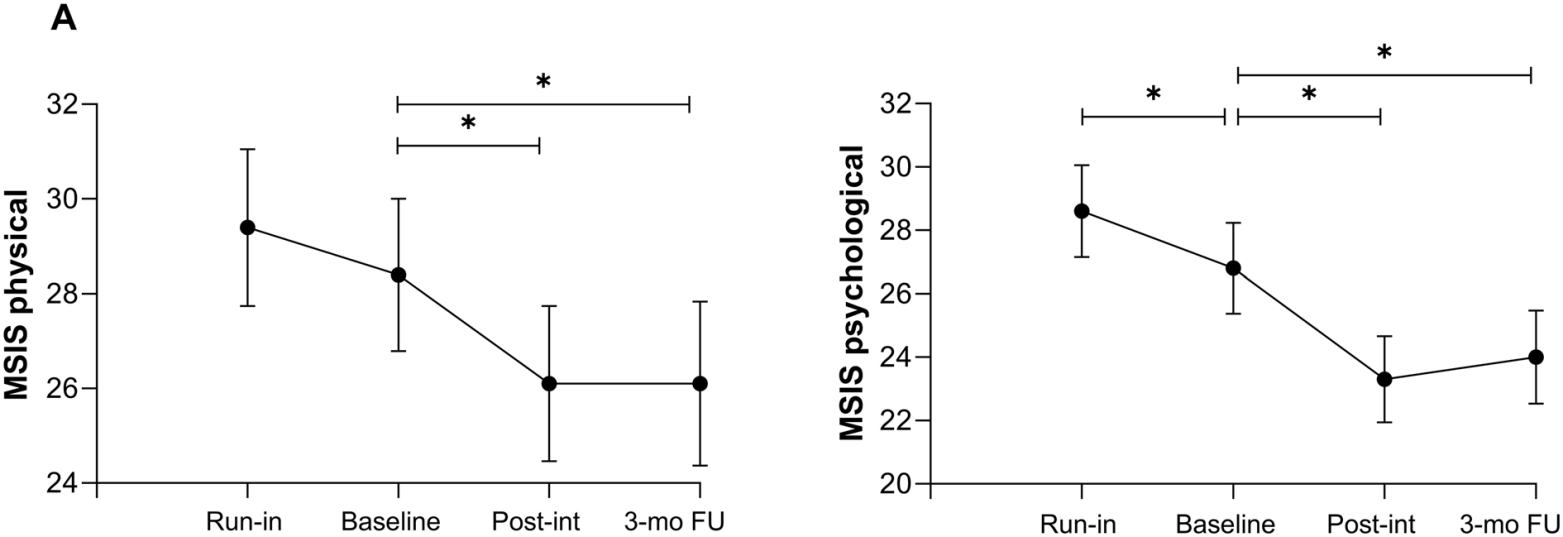
Outcome measures at each time-point. The means and 95% confidence intervals at each time-point regarding the following outcome measure: **a** MSIS-29 physical; **b** MSIS-29 psychological. *Significant change between baseline assessment and other time-points. For *p* values, see Table 2. Abbreviations: MSIS-29 = Multiple Sclerosis Impact Score; Post-int. = post-intervention; 3-mo. FU = 3-month follow-up measurement

The MSIS-29 physical score did not change from run-in to baseline, decreased from baseline to post-intervention (*β* = −2.50 [−3.40, −1.60], *P* <.001) and from baseline to 3-month follow-up (*β* = −2.00 [−2.93, −1.07], *P* <.001; Fig. 2A). MSIS-29 psychological score decreased from run-in to baseline (*β* = 1.86 [0.78, 2.94], *P* =.001), from baseline to both post-intervention (*β* = −3.48 [−4.58, −2.39], *P* <.001) and 3-month follow-up (*β* = −2.44 [−3.56, 1.31], *P* <.001; Fig. 2B). When compared to the baseline assessment, at post-intervention 21% of participants and at 3-month follow-up 20% of participants had reached an MCID of the MSIS-29 physical score (i.e., at least 8 points improvement on the MSIS physical item list) [28].

#### Secondary outcome measures

SF-12 physical scores increased from run-in to baseline (*β* = −0.86 [−1.37, −0.35], *P* =.001). No change after the intervention was noted (Supplementary Fig. 1 A). SF-12 mental scores did not change from run-in to baseline, but significantly increased from baseline to post-intervention (*β* = 1.71 [1.03, 2.40], *P* <.001). The change was not visible at 3-month follow-up (Supplementary Fig. 1B).

BMI, waist circumference, and stool type did not change from run-in to baseline, but did change from baseline to post-intervention and from baseline to 3-month follow-up (Supplementary Fig. 2). BMI decreased from baseline to post-intervention (*β* = −0.72 [−0.86, −0.58], *P* <.001) and 3-month follow-up (*β* = −0.69 [−0.83, −0.54], *P* <.001). Waist circumference reduced from baseline to post-intervention (*β* = −3.08 [−3.61, −2.56], *P* <.001) and 3-month follow-up (*β* = −2.03 [−2.57, −1.48], *P* <.001). Stool type changed from baseline to post-intervention and 3-month follow-up (*P* =.005; *P* =.003). Post hoc analyses per stool category (type 1–2, 3–4, 5–7) indicated a decrease in constipation (type 1–2; post-treatment *P* =.003; 3-month follow-up *P* =.004) and an increase in normal stool type (type 3–4; post-treatment *P* <.001; 3-month follow-up *P* =.007).

HADS anxiety score (log-transformed) significantly decreased from run-in to baseline (*β* = 0.07 [0.03, 0.11], *P* <.001), but not following the 3-month intensive intervention period (Supplementary Fig. 3 A). HADS depression (log-transformed), did not change between run-in and baseline, decreased from baseline to post-intervention (*β* = −0.09 [−0.14, −0.05], *P* <.001) and 3-month follow-up (*β* = −0.09 [−0.13, −0.04], *P* <.001; Supplementary Fig. 3B). CIS-20 subjective tiredness decreased from run-in to baseline (*β* = 0.93 [0.19,1.67], *P* =.014), and from baseline to post-intervention (*β* = −2.78 [−3.53, −2.03], *P* <.001) and 3-month follow-up (*β* = −1.74 [−2.51, −0.96], *P* <.001; Supplementary Fig. 3 C). The MSNQ-p (cognitive complaints) did not change over time (Supplementary Fig. 3D).

Based on the Mediterranean Diet Assessment Tool^36^, adherence to ingredients associated with MD did not change from run-in to baseline, improved from baseline to post-intervention (*β* = 3.62 [3.43, 3.82], *P* <.001) and 3-month follow-up (*β* = 2.76 [2.55, 2.96], *P* <.001; Supplementary Fig. 4). Adherence to the Dutch exercise guidelines remained unchanged over time (Supplementary Fig. 5). PSS (stress) scores decreased between run-in and baseline (*β* = 0.73 [0.21, 1.25], *P* =.006), from baseline to post-intervention (*β* = −1.13 [−1.66, −0.60], *P* <.001) and 3-month follow-up (*β* = −0.68 [−1.23, −0.13], *P* =.015; Supplementary Fig. 4). Regarding sleep, none of the MOS-ss subscales changed between run-in and baseline. From baseline to both post-intervention and 3-month follow-up, positive effects were identified for sleep disturbance (*β* = −5.70 [−7.02, −4.37], *P* <.001; *β* = −4.50 [−5.88, −3.13], *P* <.001, respectively), sleep adequacy (*β* = 6.04 [4.24, 7.84], *P* <.001, *β* = 3.20 [1.35, 5.06], *P* =.001, respectively), sleep somnolence (*β* = −5.16 [−6.42, −3.90], *P* <.001; *β* = −3.07 [−4.37, −1.77], *P* <.001, respectively), sleep index 1 (*β* = −4.60 [−5.67, −3.54], *P* <.001; *β* = −3.13 [−4.23, −2.03], *P* <.001, respectively), and sleep index 2 (*β* = −5.20 [−6.21, −4.19], *P* <.001; *β* = −3.46 [−4.50, −2.41], *P* <.001, respectively; Supplementary Fig. 6). The other four subscales did not change significantly (Table 2).

**Table 2:** Change of self-reported outcomes as compared to the baseline assessment

	Run-in vs. baseline		Post-intervention vs. baseline		3-month follow-up vs. baseline	
Outcomes	*β* (95%CI)	*p*	*β* (95%CI)	*p*	*β* (95%CI)	*p*
*Impact of MS on daily functioning*						
MSIS-29 physical impact scale	0.98 (0.08, 1.87)	.032^a^	**−2.50 (−3.40, −1.60)**	**<.001**^**b**^	**−2.00 (−2.93, −1.07)**	**<.001**^**b**^
MSIS-29 psychological impact scale	**1.86 (0.78, 2.94)**	**.001**^**b**^	**−3.48 (−4.58, −2.39)**	**<.001**^**b**^	**−2.44 (−3.56, 1.31)**	**<.001**^**b**^
*Quality of life*						
SF12 physical health	**−0.86 (−1.37, −0.35)**	**.001**^**b**^	0.10 (−0.42, 0.61)	.715	0.44 (−0.10, 0.97)	.108
SF12 mental health	0.15 (−0.53, 0.83)	.662	**1.71 (1.03, 2.40)**	**<.001**^**b**^	0.54 (−0.17, 1.25)	.136
*General health*						
BMI (kg/m^2^)	−0.10 (−0.24, 0.04)	.145	**−0.72 (−0.86, −0.58)**	**<.001**^**b**^	**−0.69 (−0.83, −0.54)**	**<.001**^**b**^
Waist	−0.14 (−0.66, 0.38)	.599	**−3.08 (−3.61, −2.56)**	**<.001**^**b**^	**−2.03 (−2.57, −1.48)**	**<.001**^**b**^
Stool (categorical)	−0.07 (−029, 0.15)	.536	**0.32 (0.09, 0.54)**	**.005**^**b**^	**0.34 (0.12, 0.57)**	**.003**^**b**^
*MS-specific symptoms*						
HADS anxiety (log-transformed)	**0.07 (0.03, 0.11)**	**<.001**^**b**^	−0.04 (−0.08, 0.00)	.061	−0.02 (−0.07, 0.02)	.272
HADS depression (log-transformed)	−0.002 (−0.05, 0.04)	.943	**−0.09 (−0.14, −0.05)**	**<.001**^**b**^	**−0.09 (−0.13, −0.04)**	**<.001**^**b**^
CIS-20 subjective fatigue	**0.93 (0.19, 1.67)**	**.014 **^**b**^	**−2.78 (−3.53, −2.03)**	**<.001**^**b**^	**−1.74 (−2.51, −0.96)**	**<.001**^**b**^
MSNQ-p total	0.15 (−0.33, 0.62)	.542	−0.28 (−0.76, 0.20)	.258	−0.30 (−0.80, 0.20)	.235
*Lifestyle factors*						
Nutrition | Dietary compliance score	−0.04 (−0.24, 0.15)	.682	**3.62 (3.43, 3.82)**	**<.001**^**b**^	**2.76 (2.55, 2.96)**	**<.001**^**b**^
Exercise | Moderate-to-rigorous intensity (binary)	0.34 (0.01, 0.67)	.045^a^	−0.10 (−0.43, 0.23)	.550	0.33 (−0.02, 0.67)	.062
Exercise | Strengthening (binary)	−0.36 (−0.88, 0.16)	.171	−0.14 (−0.67, 0.39)	.595	−0.52 (−1.06, 0.02)	.059
Exercise | Total (binary)	0.14 (−0.19, 0.46)	.411	−0.03 (−0.35, 0.30)	.869	0.26 (−0.08, 0.60)	.139
Stress | PSS total	**0.73 (0.21, 1.25)**	**.006**^**b**^	**−1.13 (−1.66, −0.60)**	**<.001**^**b**^	**−0.68 (−1.23, −0.13)**	**.015**^**b**^
Sleep | MOS-SS Sleep disturbance	−0.75 (−2.05, 0.56)	.264	**−5.70 (−7.02, −4.37)**	**<.001**^**b**^	**−4.50 (−5.88, −3.13)**	**<.001**^**b**^
Sleep | MOS-SS Snoring (binary)	−0.19 (−0.59, 0.20)	.340	−0.56 (−0.96, −0.15)	.007^a^	−0.04 (−0.46, 0.37)	.840
Sleep | MOS-SS SOB headache (binary)	−0.24 (−0.56, 0.08)	.137	−0.41 (−0.73, −0.08)	.013^a^	−0.11 (−0.44, 0.22)	.526
Sleep | MOS-SS Sleep adequacy	−0.09 (−1.86, 1.68)	.921	**6.04 (4.24, 7.84)**	**<.001**^**b**^	**3.20 (1.35, 5.06)**	**.001**^**b**^
Sleep | MOS-SS Sleep somnolence	1.23 (−0.01, 2.46)	.052	**−5.16 (−6.42, −3.90)**	**<.001**^**b**^	**−3.07 (−4.37, −1.77)**	**<.001**^**b**^
Sleep | MOS-SS Sleep index 1	0.25 (−0.80, 1.30)	.637	**−4.60 (−5.67, −3.54)**	**<.001**^**b**^	**−3.13 (−4.23, −2.03)**	**<.001**^**b**^
Sleep | MOS-SS Sleep index 2	−0.24 (−1.23, 0.76)	.641	**−5.20 (−6.21, −4.19)**	**<.001**^**b**^	**−3.46 (−4.50, −2.41)**	**<.001**^**b**^
Sleep | MOS-SS Sleep quantity	−0.03 (−0.12, 0.05)	.439	0.05 (−0.03, 0.14)	.224	−0.01 (−0.10, 0.08)	.841
Sleep | MOS-SS Optimal sleep (binary)	0.12 (−0.21, 0.45)	.464	0.34 (0.00, 0.68)	.050	0.40 (0.05, 0.75)	.025^a^

Intervention effects corrected for age and sex. Baseline was coded as the reference; positive values, therefore, represent a *decrease* and negative value an *increase* from run-in to baseline

**Bold** = significant after multiple comparison correction (*p* value was corrected for the number of scales analyzed per measures)

^a^*p* <.05; but not significant after multiple comparison correction

^b^*p* < s.025

*MSIS-29* Multiple Sclerosis Impact Score, *SF-12* Short-Form health survey, *BMI* *Body Mass Index*, *HADS* Hospital Anxiety and Depression Scale, *CIS-20* Checklist Individual Strength-20-r, *MSNQ-p* Multiple Sclerosis Neuropsychological Screening Questionnaire-patient version, *Exercise* adherence to the Dutch exercise guidelines, *MOS-SS* Medical Outcomes Study Sleep Scale, *SOB* shortness of breath, *PSS* Perceived Stress Scale, *Post-int.* post-intervention; *3-mo. FU* 3-month follow-up measurement

### Subgroup analysis

#### Primary outcome: impact of MS on daily functioning

The analysis on MSIS-29 was repeated in subgroups (Supplementary Table 4). Regarding the MSIS-29 physical, highly compliant participants, obese participants, and participants with low education level showed larger intervention effects at post-intervention than the total sample (*β* = −2.50), of which obese patients showed the largest effects (*β* = −5.29). The obese group also showed larger effects at 3-month follow-up (*β* = −4.32). Participants with high attendance (irrespective of MD adherence), normal weight, high education, and relapsing MS subtype showed similar intervention effects as the total sample. Finally, overweight participants and progressive MS participants showed no effects on the MSIS physical. With respect to the MSIS-29 psychological, highly compliant participants, obese participants, and participants with a low education level showed larger intervention effects at post-intervention (*β* = −5.14) and 3-month follow-up (*β* = −3.44) than the total sample (*β* = −3.48 and *β* = −2.44, respectively). Similar to the physical subscale, patients with high attendance (irrespective of MD adherence), normal weight, high education, and relapsing MS subtype showed similar intervention effects as the total sample on the psychological scale. Progressive MS patients showed similar effects at post-intervention, but this effect disappeared at 3-month follow-up. Overweight rather than obesity had no effect on the intervention outcome, a larger change was observed between run-in and baseline than between post-intervention and baseline.

#### Secondary outcomes

In the same subgroups, in addition to the primary outcome, sub-group analysis was also performed on the secondary outcomes. The results, both the description and the tables, are reported in the Supplementary results, Supplementary Tables 5 and 6.

## Discussion

This longitudinal, observational study examined the effects of the first 6-months of a 2-year online multi-modal lifestyle program with a 3-month intensive period on MS-related symptoms in 579 people with confirmed diagnosis of MS. The program was delivered by dieticians and lifestyle coaches and advocated an MD, and in addition, targeted physical activity, stress, and sleep. Following the lifestyle program was associated with reduced self-reported impact of MS on daily life as well as reduced MS-related symptoms (i.e., depressive symptoms, fatigue), improved mental QoL and general health determinants, such as BMI, waist circumference, and stool type. Additionally, the program was related to adaptations in the following targeted lifestyle domains: increased MD adherence, reduced stress levels, and improved sleep quality, but not physical activity. Overall, the effects were found to be larger in participants with higher compliance, lower education levels, and obesity.

The observational nature of the current study prevents us from drawing causal conclusions. However, the study design did allow comparison of symptom changes *after* the initiation of the lifestyle program to changes observed within the 3-month period *prior* to the intervention. Regarding the primary outcome, both the physical and psychological impact of MS on daily life decreased after the intervention, and this decrease was maintained 3 months later, although to a lesser extent. Regarding the psychological impact, a reduction was also observed in the 3 months prior to the start of the intervention, but this change was smaller than the reduction observed after intervention completion. For the physical impact of MS, a difference of ≥ 8.0 points on the MSIS-29 has been considered the MCID [28], and this was reached by 21% of the participants at post-intervention and 20% at 3-month follow-up. The small but statistically significant effect sizes are in line with a previous multi-modal lifestyle intervention in MS [23]. Altogether, our results indicate that participation in the program reduced the impact of MS on daily functioning.

Beside the reduction of the impact of MS on daily life, this program showed small positive changes on self-reported mental QoL, BMI, waist circumference, stool type, depressive symptoms, fatigue, stress (particularly in highly compliant patients, and those with normal BMI), and sleep quality, of which most effects were maintained three months later. The effects were larger in participants with higher compliance (particularly those who showed higher attendance and MD adherence), obesity and interestingly, those with a lower education level. We did not find an effect on physical QoL, anxiety, and patient-reported cognitive complaints in our total MS sample, although previous multi-modal lifestyle studies have found positive effects on these outcomes [22, 23].

To better contextualize the magnitude of change, we calculated effect sizes for continuous outcomes across time-points (Supplementary Table 7). While some outcomes, such as MSIS-29 physical and psychological scores, showed small-to-moderate effects, other outcomes—particularly those related to sleep, fatigue, and anthropometric measures—demonstrated moderate-to-large effect sizes, suggesting potentially meaningful changes.

Although current and previous observational studies, and cross-sectional data, show that adherence to a healthier diet and other lifestyle factors is associated with fewer MS symptoms, reduced physical disability, and better overall quality of life, the exact underlying mechanisms remain unknown [6, 12, 13]. To gain more insight into these mechanisms, innovative, robust and large-scale randomized-controlled trials are needed [4]. Several underlying mechanisms may apply. Mechanistic studies in animal models for MS and cross-sectional studies in MS patients have shown that diet quality is linked to disease progression and MS-related MRI outcomes, which suggests that diet may have anti-inflammatory and neuromodulating properties in MS [13, 14, 39]. Also, studies found that physical activity may modulate the neuroinflammatory and neurodegenerative processes in MS [16, 17]. It is important to realize that the presence of comorbidities, such as hypertension, diabetes, and obesity, are also associated with worse MS-disease course and are by itself associated with modifiable lifestyle factors, such as diet quality, smoking, and sedentary behavior [38, 40, 41]. This highlights the complexity of determining whether adjustments in lifestyle directly modulate the MS pathogenesis, indirectly modulate the MS pathogenesis by modulating comorbidities or both. In addition to mechanistic explanations and probably placebo effect, being a member of an online community aiming to improve MS-related symptoms may strengthen the feelings of wellbeing, self-management skills, and empowerment, independent of the content of the program.

The current study design (i.e., an online, multi-domain lifestyle program) has advantages and limitations. The advantages include the combination of an online format and a design that ensured all participants could participate in the lifestyle program, which facilitated the inclusion of a large and diverse sample. The use of a run-in period allowed us to observe within-subject changes before and after the intervention, providing a valuable comparison in the absence of a randomized control group. While the lack of randomization and blinding limit internal validity, the pragmatic nature of the design reflects real-world clinical practice and supports external validity. Furthermore, our design enables flexibility, personalization, and scalability—key considerations in implementing sustainable lifestyle interventions. In addition, all participants had a confirmed diagnosis of MS and there was a reasonable attendance rate [42]. Limitations include the generalizability toward the whole MS community. Regarding the patients who dropped out, we recorded that 56% had never smoked in the analyzed sample compared to 45% in the drop-out group. Since smoking may also be a marker of a healthier lifestyle in general, it may suggest that the participants who dropped out are less healthy and less motivated to modify their lifestyle. However, this is an important challenge in general when considering lifestyle modification studies or their application in clinical practice. Furthermore, the majority of study population was female, which can also affect the generalizability. Due to the nature of the study design (i.e., no physical appointments for measurements), we had to rely on self-reported questionnaires and measures. Consequently, responses may be affected by social desirability, inaccuracy of self-evaluation, or recall bias, especially since the intervention did not allow blinding. Future studies will benefit from adding objective outcome measures, including a neurological examination by an examiner who is blinded to the intervention status of the patient, MRI scans, and blood and digital biomarkers. Although the 3-month run-in period served as a possibility to assess changes over time without the program offered, a randomized-controlled trial is needed for causal conclusions regarding intervention effects and to assess the contributing factor of placebo effect. This is particularly important given the improvements observed during the run-in period, which may have been influenced by anticipatory effects or natural fluctuations, potentially overshadowing the true effects of the intervention. Compliance was defined as a combination of attendance and MD adherence. Unfortunately, we had no data whether patients were compliant with the advice given on physical activity, relaxation, and sleep. It would be relevant to monitor adherence to each lifestyle component in future studies, for example with wearables, so that the effect of each individual component could be assessed accordingly. Apart from these limitations, this study is considered replicable as the program was delivered online with a fixed curriculum. This design allows a heterogeneous group of MS patients, including those with a higher level of disability or those living in areas with less access to health care, to participate.

In conclusion, this study offered a unique investigation of a digital multi-modal lifestyle intervention in a large sample of people with MS, and results suggest that this intervention might lead to beneficial effects on the physical and psychological impact of MS on daily life, mental QoL, MS-specific symptoms, and general health. These effects are promising. Moreover, lifestyle adaptations are rarely accompanied with side-effects and supports a healthy lifestyle in general. However, well-designed randomized trials—combined with biomarkers to assess underlying mechanistic changes over time—are needed to determine clinically important effects from lifestyle adaptations in addition to pharmacological treatments of MS.

## Supplementary Information

Below is the link to the electronic supplementary material.

Supplementary file1 (DOCX 665 KB)

## Data Availability

Anonymized data, not published in the article, can be shared upon reasonable request from a qualified investigator.
