# Supplementary material for: A multi-domain lifestyle intervention in multiple sclerosis: a longitudinal observational study
Source: J Neurol. 2025 Jun 24;272(7):476. doi: 10.1007/s00415-025-13196-9 (PMC12187891; doi:10.1007/s00415-025-13196-9)
Supplement: Supplementary file 1 — Supplementary file1 (DOCX 665 KB) [file 415_2025_13196_MOESM1_ESM.docx]

**Supplementary Material**

1. Supplementary Table 1
2. Supplementary Table 2
3. Supplementary Methods
4. Supplementary Table 3
5. Supplementary Table 4
6. Supplementary Figure 1
7. Supplementary Figure 2
8. Supplementary Figure 3
9. Supplementary Figure 4
10. Supplementary Figure 5
11. Supplementary Figure 6
12. Supplementary Results
13. Supplementary Table 5
14. Supplementary Table 6
15. Supplementary Table 7

**Supplementary Table 1. Content of the main meetings and theme hours of the online lifestyle intervention, including their timings in weeks**

| **Meeting** | **Topic** | **Brief content description** |
| --- | --- | --- |
| Introduction  (t=-1) | Introduction online system | Instructions about lifestyle program, online platform and e-learning environment and expectations. |
| Main meeting 1 (t=0) | Nutrition | Information on nutrition, session with dietician, session with lifestyle coach about intrinsic motivation |
| Theme hour 1 (t=2) | Nutrition / How is it going? | Recap nutrition, group conversations about participant progress with focus on awareness, self-care, self-acceptance and taking responsibility. |
| Theme hour 2 (t=3) | How is it going? | Group conversation about participant progress with focus on awareness, self-care, self-acceptance and taking responsibility |
| Main meeting 2 (t=4) | Physical activity | Relaxation exercises, recap nutrition, interim low-effort physical exercises, information on physical activity, sub-session with dietician with advice on how to personalize/transform recipes including how to read labels, session with physical activity coach. |
| Theme hour 3 (t=6) | Physical activity / How is it going? | Recap physical activity, group conversation about participant progress with focus on awareness, self-care, self-acceptance and taking responsibility. |
| Theme hour 4 (t=8) | Relaxation | Round off physical activity, information on relaxation, relaxation exercises. |
| Theme hour 5  (t=10) | Relaxation / How is it going? | Recap relaxation, group conversation about participant progress with focus on awareness, self-care, self-acceptance and taking responsibility. |
| Main meeting 3  (t=12)  From main meeting 3 onward meetings (up to 24 months) | Sleep      Session every 3 months on one of the pillars or coaching | Recap relaxation, information on sleep, interim low-effort physical exercises, introduction inspiration trajectory, coaching session including reflection on the past three months/  In order to stay motivated and maintain the behavior change on a longer term, we offer facultative sessions to the participants with changing topics. We do not offer new information during this phase, but rather ‘refresh’ their memory and tackle obstacles. |

**Supplementary Table 2. Dietary recommendations for a modified Mediterranean diet**

| **Daily recommendations** | **Weekly recommendations** |
| --- | --- |
| Use extra virgin olive oil as main fat source | Consume as little as possible processed meat |
| Consume more than four tablespoons of olive oil | Consume a maximum of three portions (300 grams) of unprocessed poultry |
| Consume more than seven serving spoons (350 grams) of vegetables, preferably a mix of colors | Consume a maximum of three portions (300 grams) of unprocessed red meat |
| Consume two to three pieces of fruit | Consume as little as possible alcohol |
| Consume as little as possible margarine or light cream | Consume at least three portions (300 grams) of legumes |
| Consume as little as possible (sugar) sweetened beverage e.g. soda and fruit juice | Consume no sweet or savory snacks (e.g. cake, chips) |
| Consume all food in three eating moments throughout the day | Consume at least three (90 grams) portions of unroasted, unsalted nuts (including peanuts and seeds) |
|  | Consume at least two portions (200 grams) of fish or seafood |
|  | Consume unprocessed whole fat dairy (fermented if possible) and as little as possible skimmed or low-fat dairy |
|  | Consume whole grain products and as little as possible refined grain products |

**Supplementary Methods – Lifestyle Intervention**

Participants attended three main meetings and five theme hours. The main meetings were designed for knowledge dissemination in groups of 100 participants and also included activities in smaller groups of 25 or less. The main meetings had a duration of five hours. The theme hours were interim shorter meetings in fixed groups of approximately 25 participants. These groups were identical to those during the activities in the main meetings. The theme hours consisted of conversations between participants regarding their behavioral changes and experiences with the program, centered around the overarching themes awareness, self-care, self-acceptance and taking responsibility. For each pillar (nutrition, exercise, relaxation and sleep) participants were provided with comprehensible knowledge and practical exercises and they were asked to actively experience the lifestyle changes and reflect on them. The interventions and information during the program were provided in a standardized manner using pre-defined protocols.

**Online platform**

During the program days all information with respect to one pillar is explained and discussed. After the program day, all information can be found on the online platform as well in different forms (video, text, animation, slideshow, etc.). Participants had access to an online community, which enabled contact with other participants or the support team to pose questions and exchange advice, motivation, experiences or pictures. Furthermore, an e-learning environment was created comprising amongst others background information, knowledge videos, assignments, podcasts, challenges and recipes.

**Diet**

To teach participants how to prepare food in accordance with the study dietary guidelines, the online platform offered a complete dietary program, including dietary advice, recipes and menus for 28 days. Participants were responsible for buying and cooking their own food. From the second month onward participants had to find or create their own recipes that complied with the study dietary guidelines. Therein, participants were guided online by dieticians.

**Physical activity**

Lifestyle coaches guided participants online with their personal physical activity plan during a group session. The main goal was to increase physical activity in each individual, regardless of their level of physical fitness at the time.^1^ Additionally, for a month, daily, podcasts, videos or blogs on physical activity were published on the online platform. Various exercises were provided focusing on strength, flexibility, endurance, speed and coordination. Adjustments were available for participants in a wheelchair. The podcasts addressed various themes, posed questions and provided assignments. The videos provided general, flexibility and muscle strength exercises.

**Stress**

Exercises for stress relief were posted on the online platform with a focus on breathing, positivity, gratitude and mindfulness to address physical, mental, emotional and behavioral complaints.

**Sleep**

Lastly, tips and exercises were published on the online platform daily to improve sleep quality. This is based on what is currently known about sleep hygiene in literature. For example, to regulate sleeping room temperature or be cautious with consuming caffeine in the afternoon or evening or eating food shortly before going to bed. Thereafter, participants could decide what would be personally beneficial and if it was necessary for them to make changes.

1. Weggemans RM, Backx FJG, Borghouts L, et al. The 2017 Dutch Physical Activity Guidelines. Int J Behav Nutr Phys Act 2018;15:58.

**Supplementary Table 3. Outcome measures at each time-point**

|  | Run-in (*n*=597) | | Baseline (*n*=597) | | Post-int. (*n*=567) | | 3-mo. FU (*n*=520) | |
| --- | --- | --- | --- | --- | --- | --- | --- | --- |
| ***Impact of MS on daily functioning*** |  |  |  |  |  |  |  |  |
| MSIS-29 physical impact scale^b^ | 29.4 | 20.3 | 28.4 | 19.7 | 26.1 | 19.7 | 26.1 | 19.9 |
| MSIS-29 psychological impact scale^b^ | 28.6 | 17.8 | 26.8 | 17.6 | 23.3 | 16.4 | 24.0 | 16.9 |
| ***Quality of life*** |  |  |  |  |  |  |  |  |
| SF12 physical health^b^ | 40.1 | 9.3 | 40.9 | 9.1 | 41.0 | 9.0 | 41.3 | 9.3 |
| SF12 mental health^b^ | 51.0 | 9.1 | 50.8 | 9.7 | 52.5 | 9.3 | 51.5 | 9.3 |
| ***General health*** |  |  |  |  |  |  |  |  |
| BMI (kg/m2)^b^ | 25.6 | 4.5 | 25.6 | 4.6 | 24.9 | 4.6 | 25.0 | 4.5 |
| Waist (cm)^b^ | 92.0 | 13.3 | 92.1 | 13.0 | 89.0 | 12.3 | 90.1 | 12.8 |
| Stool (type I/II/III/IV/V/VI/VII)^d^ | 10/18/23/39/7/4/0% | | 8/17/26/37/8/4/0 | | 6/14/23/49/6/3/1 | | 6/14/24/47/8/2/0 | |
| ***MS specific symptoms*** |  |  |  |  |  |  |  |  |
| HADS anxiety^a^ | 5.0 | 5.0 | 5.0 | 5.0 | 5.0 | 4.0 | 5.0 | 4.0 |
| HADS depression^a^ | 4.0 | 6.0 | 4.0 | 5.0 | 3.0 | 5.0 | 3.0 | 5.0 |
| CIS-20 subjective fatigue^b^ | 36.0 | 11.2 | 35.1 | 11.7 | 32.4 | 12.1 | 33.1 | 12.2 |
| MSNQ-p total cognitive complaints^b^ | 22.7 | 10.1 | 22.5 | 10.0 | 22.3 | 10.1 | 22.0 | 10.3 |
| ***Lifestyle factors*** |  |  |  |  |  |  |  |  |
| Dietary compliance score^b^ | 7.1 | 2.2 | 7.1 | 2.1 | 10.7 | 2.4 | 9.9 | 2.4 |
| Exercise \| Total | 278 | 48% | 269 | 47% | 255 | 46% | 251 | 50% |
| Exercise \| Moderate-to-vigorous | 310 | 54% | 287 | 50% | 267 | 48% | 271 | 54% |
| Exercise \| Strengthening | 483 | 83% | 495 | 86% | 472 | 85% | 419 | 84% |
| PSS total stress^b^ | 19.9 | 8.4 | 19.2 | 8.7 | 18.1 | 8.5 | 18.3 | 8.6 |
| MOS-SS Sleep disturbance | 34.0 | 21.8 | 34.7 | 22.1 | 29.2 | 19.9 | 29.8 | 20.9 |
| MOS-SS Snoring (yes)^c^ | 323 | 56% | 333 | 58% | 292 | 53% | 286 | 57% |
| MOS-SS Short of breath, headache (yes)^c^ | 226 | 39% | 245 | 42% | 203 | 37% | 199 | 40% |
| MOS-SS Sleep adequacy | 46.0 | 22.0 | 46.1 | 26.1 | 52.0 | 25.7 | 50.5 | 26.8 |
| MOS-SS Sleep somnolence | 34.2 | 22.0 | 32.9 | 21.9 | 28.0 | 20.3 | 29.4 | 20.9 |
| MOS-SS Sleep index 1 | 37.0 | 16.8 | 36.7 | 17.2 | 32.3 | 16.4 | 32.9 | 17.4 |
| MOS-SS Sleep index 2 | 36.7 | 16.4 | 36.9 | 17.1 | 31.9 | 15.9 | 32.8 | 16.8 |
| MOS-SS Sleep quantity | 7.2 | 1.1 | 7.3 | 1.4 | 7.3 | 1.1 | 7.3 | 1.0 |
| MOS-SS Optimal sleep (yes)^c^ | 371 | 64% | 362 | 63% | 366 | 66% | 341 | 68% |

^a^Median (IQR), ^b^mean (SD), ^c^n (%), ^d^%. Abbreviations: MSIS-29 = Multiple Sclerosis Impact Score; SF-12 = Short Form health survey; BMI = Body Mass Index; HADS = Hospital Anxiety and Depression Scale; CIS-20 = Checklist Individual Strength-20-r; MSNQ-p = Multiple Sclerosis Neuropsychological Screening Questionnaire – patient version; Exercise = adherence to the Dutch exercise guidelines; MOS_SS = Medical Outcomes Study Sleep Scale; SOB = shortness of breath; PSS = Perceived Stress Scale; Post int. = Post-intervention; 3-mo. FU= 3-month follow-up measurement**.**

**Supplementary Table 4. Intervention effects on the primary outcome (MSIS-29) in subgroups**

|  |  | **MSIS-29 physical** | | | **MSIS-29 psychological** | | |
| --- | --- | --- | --- | --- | --- | --- | --- |
|  |  | Run-in  vs. baseline* | Post-intervention vs. baseline | 3-month follow-up vs. baseline | Run-in  vs. baseline* | Post-intervention vs. baseline | 3-month follow-up vs. baseline |
| **Total** | Full sample (*n*=597) | 0.98 (0.08, 1.87)^ | **-2.50 (-3.40, -1.60)**** | **-2.00 (-2.93, -1.07)**** | **1.86 (0.78, 2.94)*** | **-3.48 (-4.58, -2.39)**** | **-2.44 (-3.56, 1.31)**** |
| **Compliance** | Attendance (*n*=355) | **1.63 (0.49, 2.77)*** | **-2.91 (-4.06, -1.77)**** | **-1.98 (-3.16, -0.81)*** | **1.99 (0.68, 3.29)*** | **-3.94 (-5.25, -2.64)**** | **-2.79 (-4.14, -1.44)**** |
|  | Attendance and MD^a^ (*n*=159) | 1.78 (0.05, 3.50)^ | **-3.93 (-5.66, -2.21)**** | **-2.14 (-3.92, -0.36)*** | 1.69 (-0.26, 3.65) | **-5.14 (-7.09, -3.18)**** | **-4.03 (-6.04, -2.01)**** |
|  | Attendance and ΔMD^b^ (*n*=157) | 1.88 (0.22, 3.54)^ | **-3.61 (-5.27, -1.95)**** | **-2.13 (-3.85, -0.41)*** | 2.05 (0.14, 3.97) | **-5.79 (-7.70, -3.87)**** | **-3.92 (-5.91, -1.93)**** |
| **BMI** | Normal weight (*n*=288) | 0.49 (-0.75, 1.73) | **-2.41 (-3.67, -1.15)**** | -1.39 (-2.68, -0.10)^ | 0.58 (-0.97, 2.14) | **-3.16 (-4.74, -1.58)**** | **-2.50 (-4.12, -0.89)*** |
|  | Overweight (*n*=174) | **1.90 (0.27, 3.54)*** | -1.34 (-3.00, 0.31) | -1.92 (-3.62, -0.22^ | **3.80 (1.90, 5.71)**** | **-2.64 (-4.57, -0.71)*** | -1.99 (-3.98, -0.003) |
|  | Obese (*n*=104) | 0.43 (-1.65, 2.52) | **-5.29 (-7.38, -3.20)**** | **-4.32 (-6.51, -2.13)**** | 1.90 (-0.56, 4.36) | **-5.90 (-8.37, -3.44)**** | **-3.44 (-6.02, -0.86)*** |
| **Education** | Low education (*n*=184) | 0.92 (-0.76, 2.61) | **-3.52 (-5.22, -1.81)**** | **-2.81 (-4.58, -1.04)*** | **2.91 (0.86, 4.96)*** | **-5.26 (-7.33, -3.19)**** | **-3.56 (-5.51, -1.20)*** |
|  | High education (*n*=395) | 1.00 (-0.04, 2.04) | **-2.02 (-3.08, -0.96)**** | **-1.62 (-2.71, -0.54)*** | 1.36 (0.11, 2.62)^ | **-2.65 (-3.93, -1.38)**** | **-2.02 (-3.33, -0.71)*** |
| **MS type** | Relapsing MS (*n*=411) | 0.83 (-0.19, 1.85) | **-2.62 (-3.66, -1.59)**** | **-2.27 (-3.34, -1.19)*** | **1.83 (0.54, 3.12)*** | **-3.31 (-4.62, -2.01)**** | **-2.63 (-3.98, -1.28)**** |
|  | Progressive MS (*n*=124) | 2.03 (0.06, 4.00)^ | -1.22 (-3.20, 0.76) | -0.40 (-2.44, 1.64) | 1.55 (-0.58, 3.67) | **-3.26 (-5.40, -1.11)*** | -1.34 (-3.54, 0.87) |

*Note.* The Table presents the intervention effects corrected for age and gender, represented by β(95%CI), per subgroup and time-point. Bold=significant after multiple comparison correction. ^*p*<.05, but not significant after multiple comparison correction, **p*<.025 (significant)*, **p*<.001 (significant)*.* ^a^Baseline was coded as the reference; positive values therefore represent a *decrease* and negative values an *increase* from run-in to baseline. ^b^MD represents the upper tertile of patients regarding their level of MD compliance at post-intervention. ^c^ΔMD indicates the upper tertile of patients regarding their change in MD between baseline and post-intervention. Abbreviations: MSIS-29 = Multiple Sclerosis Impact Score; MD = Mediterranean Diet; BMI = Body Mass Index; MS= multiple sclerosis; Post int. = Post-intervention; 3-mo. FU = 3-month follow-up measurement**.**

**
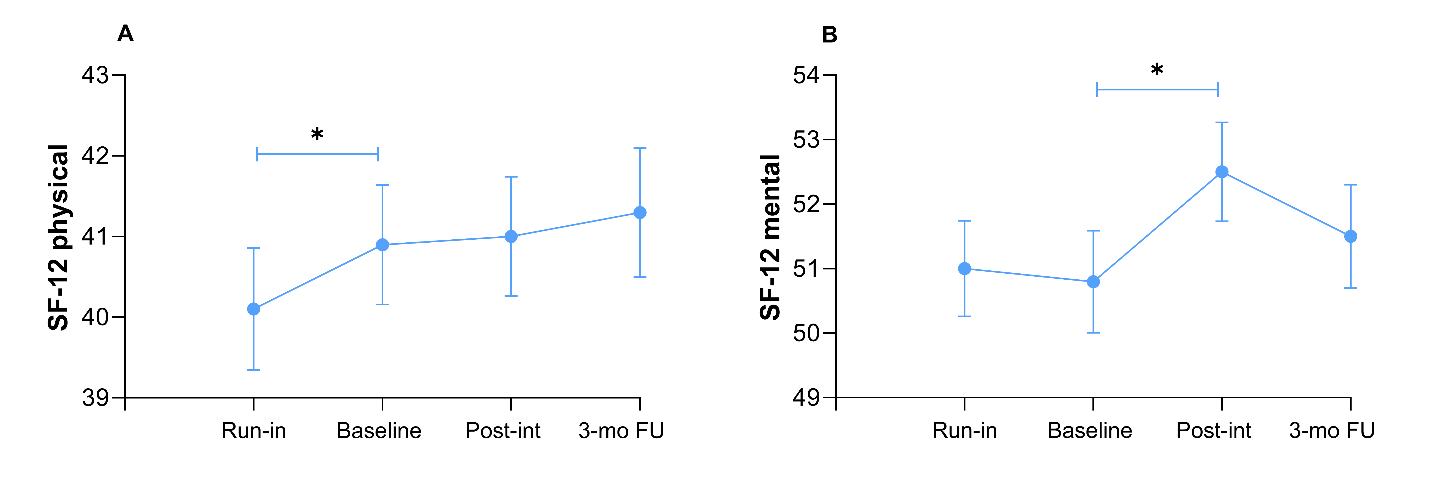
**

**Supplementary Figure 1**. **Outcome measures at each time-point**

The means and 95% confidence intervals at each time-point regarding the following outcome measure: (a) SF-12 physical; (b) SF-12 mental. *significant change between baseline assessment and other time-points. For p-values see Table 2. Abbreviations: SF-12 = Short Form health survey; Post int. = Post-intervention; 3-mo. FU = 3-month follow-up measurement.

**
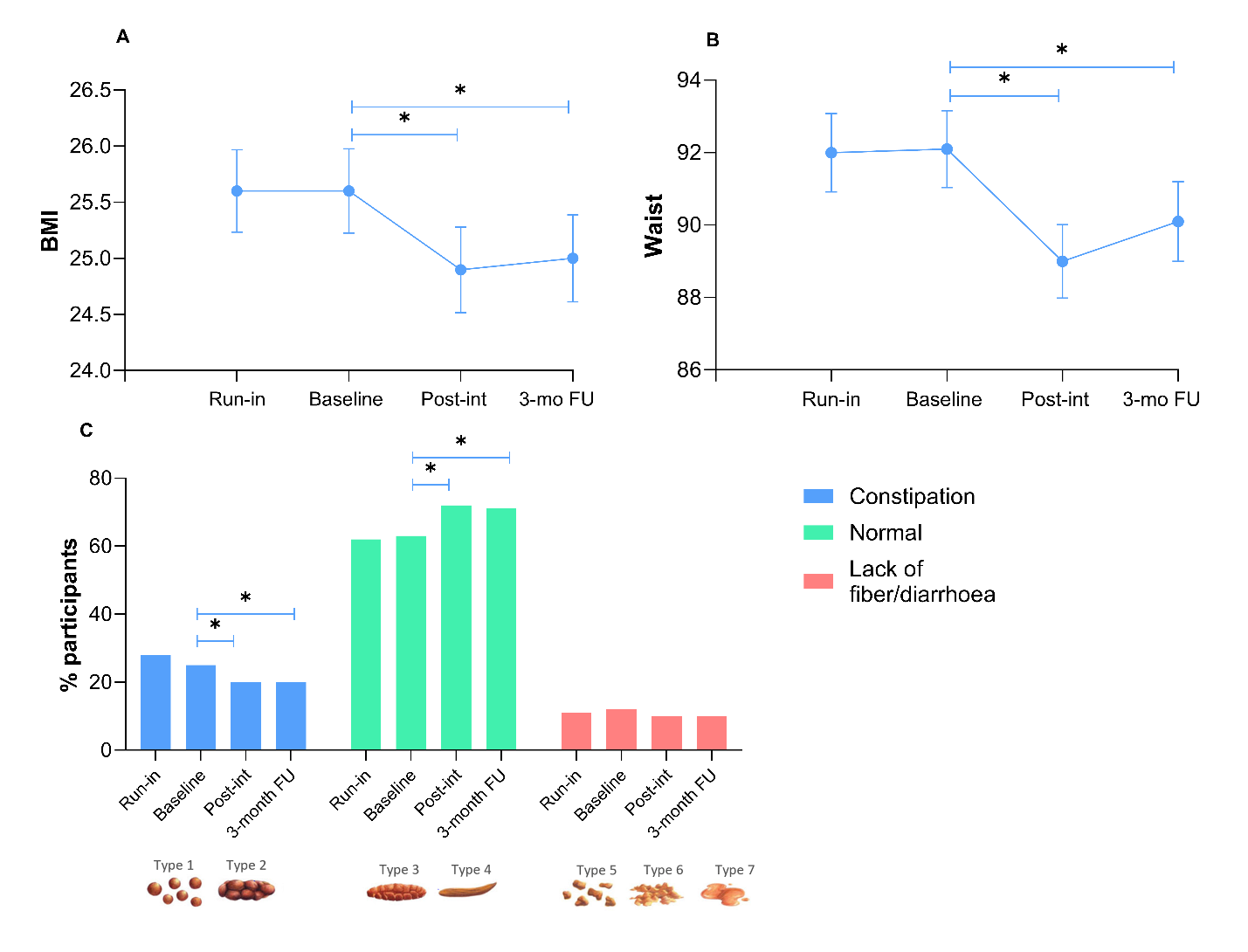
**

**Supplementary Fig 2** **Intervention effects on general health**

**(a)** The means and 95% confidence intervals at each time-point regarding BMI; **(b)** The means and 95% confidence intervals at each time-point regarding waist circumference; **(c)** The percentage of participants with a specific stool type (i.e. determined by the Bristol Stool Chart) per time-point. *significant change between baseline assessment and other time-points. For p-values see Table 2. Abbreviations: BMI=body mass index; Post int. = Post-intervention; 3-mo. FU = 3-month follow-up measurement**.**

**
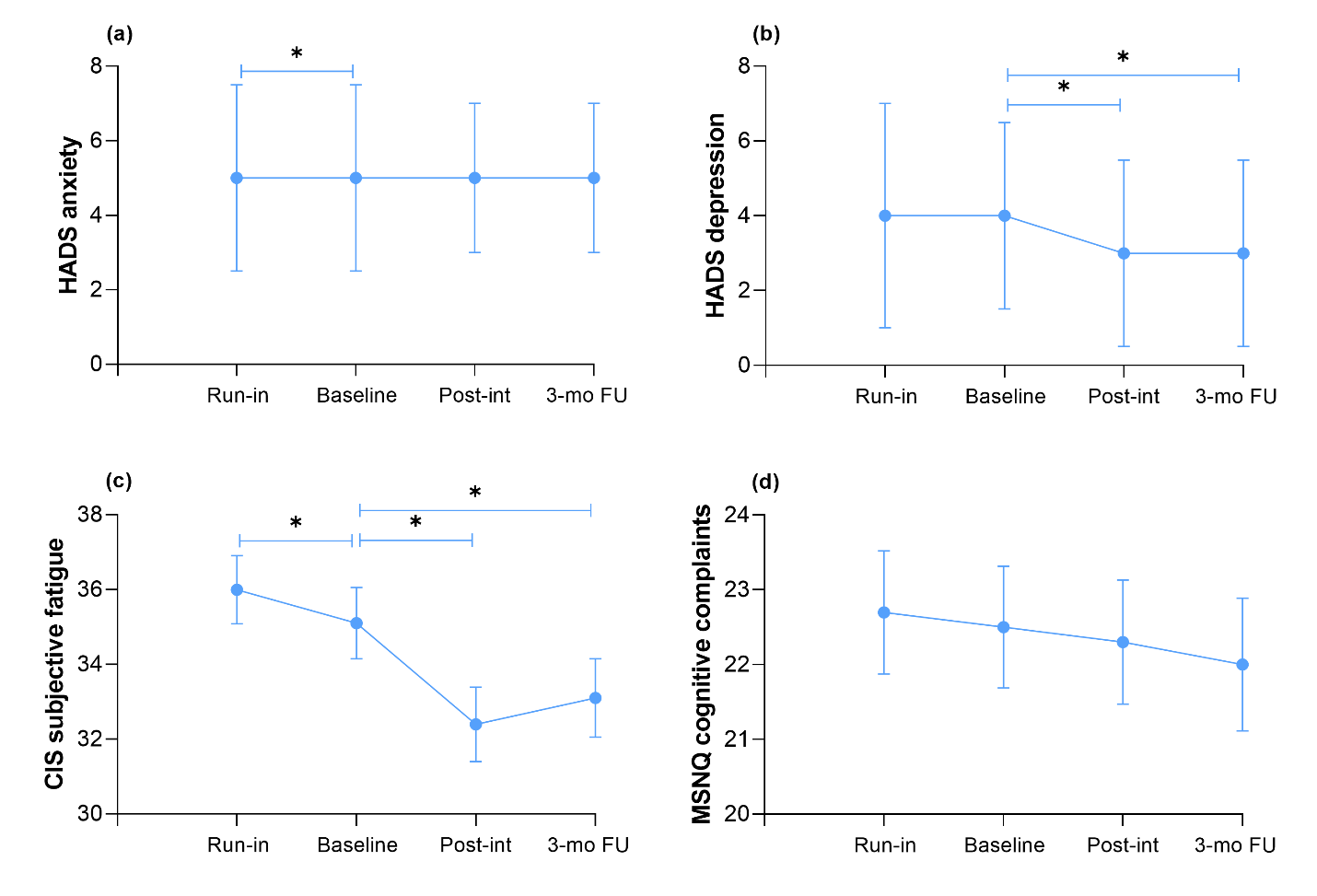
**

**Supplementary Fig 3** **Intervention effects on MS-specific symptoms**

**(a)** The median and IQR/2 at each time-point regarding HADS anxiety level; **(b)** The median and IQR/2 at each time-point regarding HADS depression level; **(c)** The means and 95% confidence intervals at each time-point regarding CIS subjective fatigue; **(d)** The means and 95% confidence intervals at each time-point regarding MSNQ-P subjective cognitive complaints. *significant change between baseline assessment and other time-points. Notice, HADS scores were log-transformed before used in the analysis. For p-values used see Table 2. Abbreviations: HADS = Hospital Anxiety and Depression Scale; CIS-20 = Checklist Individual Strength-20-r; MSNQ-p = Multiple Sclerosis Neuropsychological Screening Questionnaire – patient version; Post int. = Post-intervention; 3-mo. FU = 3-month follow-up measurement; IQR = Interquartile Range**.**

**
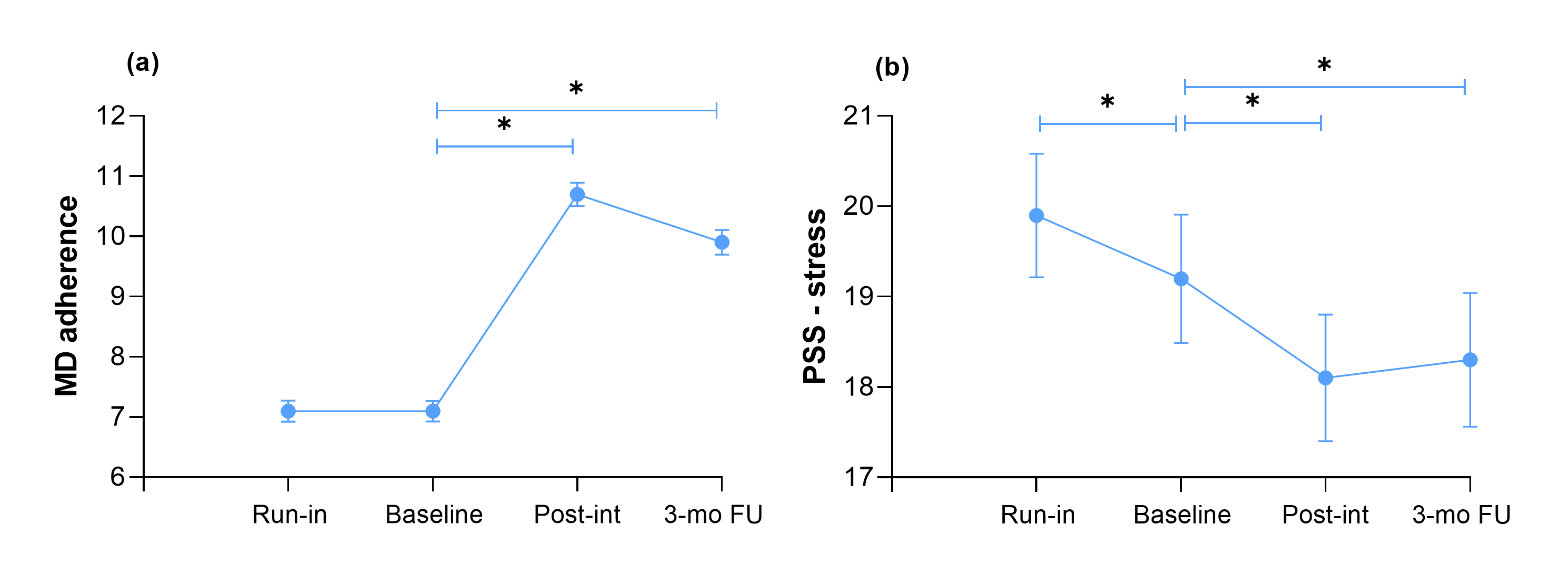
**

**Supplementary Fig 4:** **Intervention effects on dietary compliance and stress**

The means and 95% confidence intervals at each time-point regarding the following outcome measure: (a) MD dietary adherence; (b) PSS stress total score. Abbreviations: MD = Mediterranean diet; PSS = Perceived Stress Scale; Post int. = post-intervention; 3-mo. FU = 3-month follow-up measurement**.**

**
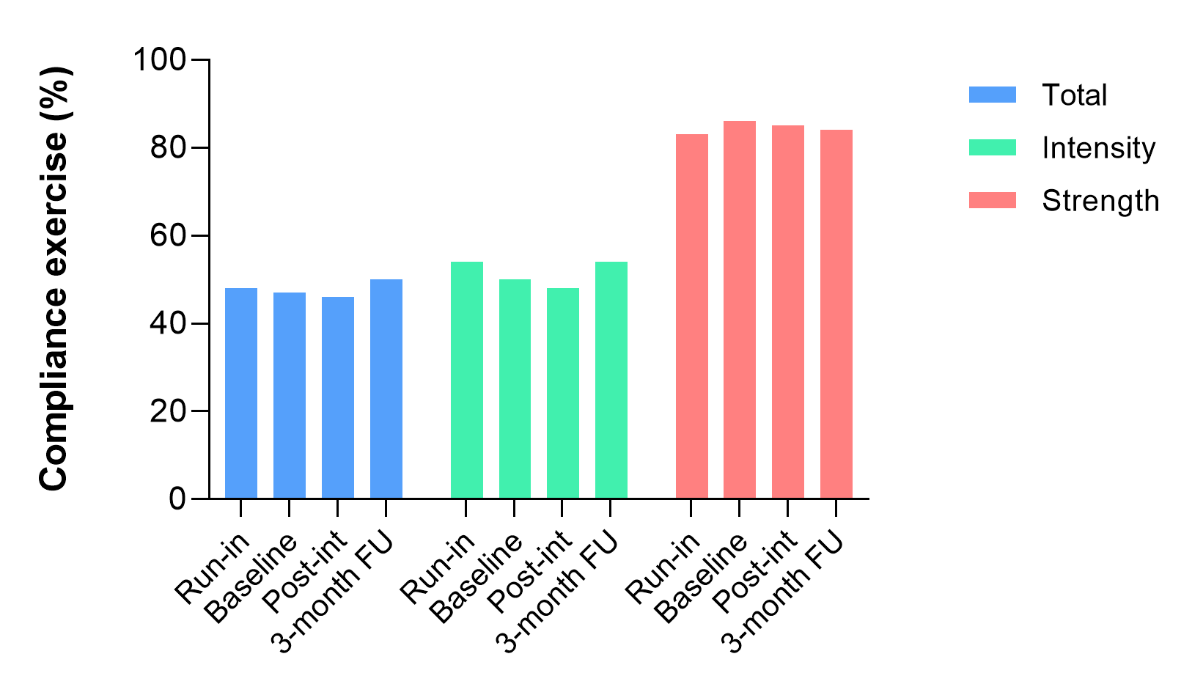
**

**Supplementary Fig 5**. **Intervention effects on exercise compliance**

The percentage of patients that comply with the exercise guidelines. Abbreviations: Post int. = Post-intervention; 3-mo. FU = 3-month follow-up measurement**.**


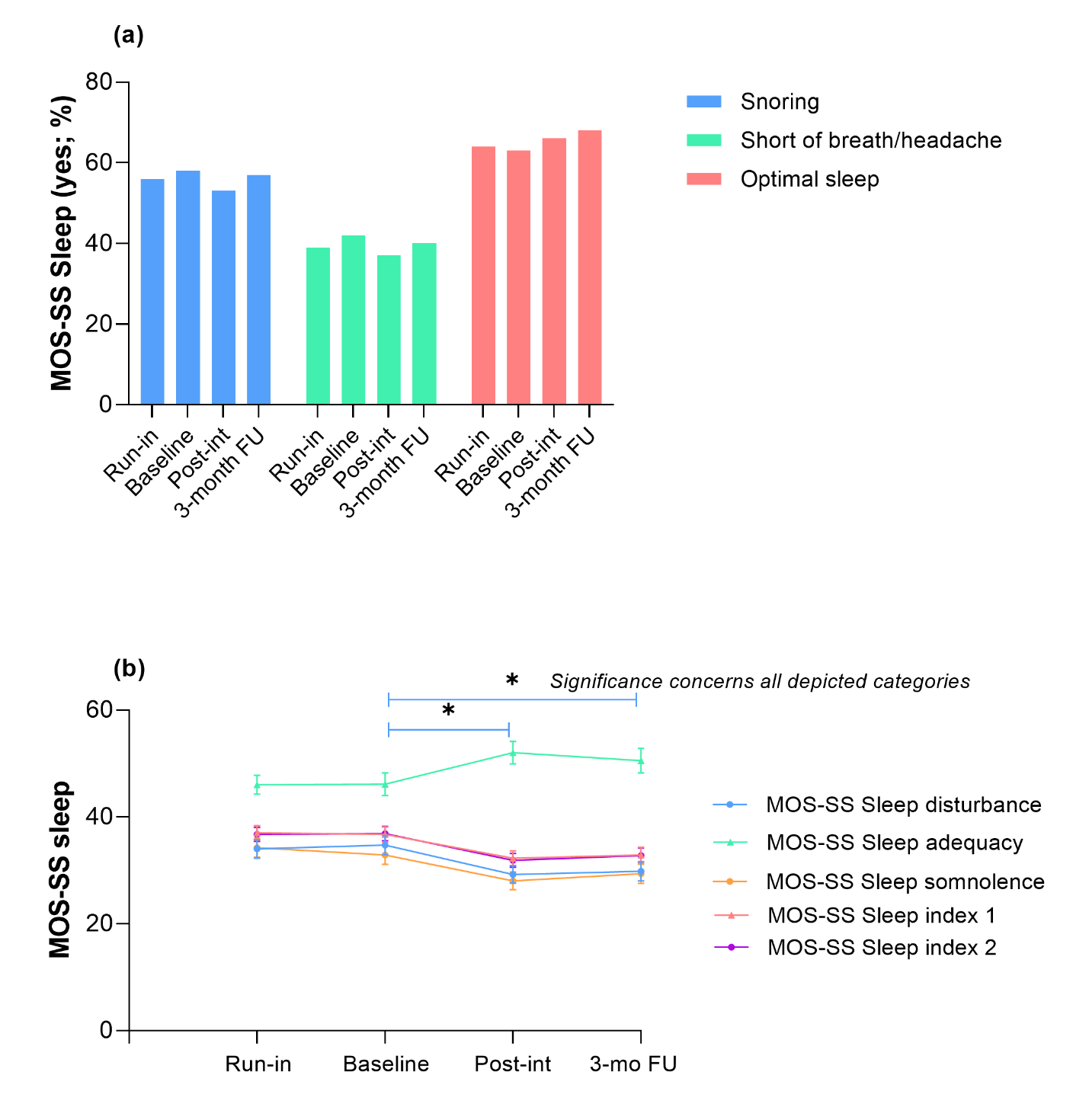


**Supplementary Fig 6**. **Intervention effects on sleep**

(a) The percentage of patients that snore (blue), are short of breath or have a headache during sleep (green), and who consider their sleep optimal (red) per time-point; (b) The means and 95% confidence intervals at each time-point regarding MOS-SS subscales. Abbreviations: MOS-SS = Medical Outcomes Study Sleep Scale; Post int. = Post-intervention; 3-mo. FU = 3-month follow-up measurement**.**

**Supplementary Results**

**Subgroup analyses on secondary outcomes**

**Compliance groups**

Results can be found in Supplementary Tables 4 and 5. Regarding compliance, an effect on HADS anxiety was found in the high attendance group: the anxiety level remained stable from run-in to baseline (*p*>.025) yet declined from baseline to post-intervention (β=-0.06, *p*=.013), which was not found 3 months later (*p*>.025). Regarding cognitive complaints (MSNQ-p), the group with high attendance and high MD adherence at post-intervention remained stable from baseline to run-in and post-intervention but did show a reduction from baseline to 3-month follow-up (β=-1.10, *p*=.021). Regarding stress (PSS), the high compliant groups (combination of attendance and MD) remained stable in the run-in period (contrasting the attendance group and total sample), whereas their stress level decreased from baseline to post-intervention, and in one group (i.e. attendance and ΔMD) also three months later.

**BMI groups.**

SF-12 mental did not significantly increase within the overweight sample from baseline to post-intervention, whereas the normal (β=1.87, *p*<.001) and obese (β=2.92, *p*<.001) groups did show an increase, similar to the whole sample. Stool showed no effect in the normal weight (i.e. change before and after intervention was similar) and obese samples, whereas it did show an effect after the intervention in the overweight sample (β=0.43, *p*=.038). In contrast to the other groups, the obese group did show a reduction in cognitive complaints (MSNQ-p) from baseline to post-intervention (β=-1.27, *p*=.023), while no changes were found from run-in to baseline and baseline to 3-month follow-up. The normal weight sample showed a reduction in adherence to the strengthening guidelines from baseline to 3-month follow-up (β=-1.00, *p*=.014), whereas the other time-periods and samples remained stable. The normal weight group showed a reduction in stress (PSS) from baseline to post-intervention (β=-1.38, *p*=.001), and not during the other time-periods, nor did the overweight or obese group show reductions in stress after the intervention.

|  |  | **Attendance (n=355)** | | **Attendance and MD post int. (n=159)** | | **Attendance and ΔMD (n=157)** | |
| --- | --- | --- | --- | --- | --- | --- | --- |
| **Outcomes** |  | **β (95%CI)** | ***p*** | **β (95%CI)** | ***p*** | **β (95%CI)** | ***p*** |
| MSIS-29 physical | Run-in vs. baseline | **1.63 (0.49, 2.77)** | **.005** | 1.78 (0.05, 3.50) | .043^a^ | 1.88 (0.22, 3.54) | .027^a^ |
|  | Post-int. vs. baseline | **-2.91 (-4.06, -1.77)** | **<.001** | **-3.93 (-5.66, -2.21)** | **<.001** | **-3.61 (-5.27, -1.95)** | **<.001** |
|  | 3-month FU vs. baseline | **-1.98 (-3.16, -0.81)** | **.001** | **-2.14 (-3.92, -0.36)** | **.018** | **-2.13 (-3.85, -0.41)** | **.015** |
| MSIS-29 psychological | Run-in vs. baseline | **1.99 (0.68, 3.29)** | **.003** | 1.69 (-0.26, 3.65) | .089 | 2.05 (0.14, 3.97) | .036^a^ |
|  | Post-int. vs. baseline | **-3.94 (-5.25, -2.64)** | **<.001** | **-5.14 (-7.09, -3.18)** | **<.001** | **-5.79 (-7.70, -3.87)** | **<.001** |
|  | 3-month FU vs. baseline | **-2.79 (-4.14, -1.44)** | **<.001** | **-4.03 (-6.04, -2.01)** | **<.001** | **-3.92 (-5.91, -1.93)** | **<.001** |
| SF12 physical health | Run-in vs. baseline | **-1.22 (-1.87, -0.56)** | **<.001** | **-1.49 (-2.45, -0.52)** | **.002** | **-1.25 (-2.16, -0.33)** | **.007** |
|  | Post-int. vs. baseline | 0.03 (-0.62, 0.69) | .925 | 0.53 (-0.43, 1.49) | .282 | 0.04 (-0.87, 0.96) | .923 |
|  | 3-month FU vs. baseline | 0.32 (-0.35, 1.00) | .344 | 0.46 (-0.53, 1.44) | .366 | 0.56 (-0.38, 1.50) | .245 |
| SF12 mental health | Run-in vs. baseline | 0.40 (-0.45, 1.24) | .356 | 0.69 (-0.59, 1.98) | .291 | 0.63 (-0.61, 1.87) | .317 |
|  | Post-int. vs. baseline | **2.39 (1.55, 3.23)** | **<.001** | **2.80 (1.52, 4.09)** | **<.001** | **3.67 (2.44, 4.91)** | **<.001** |
|  | 3-month FU vs. baseline | 0.66 (-0.21, 1.53) | .137 | 0.83 (-0.49, 2.14) | .220 | 1.18 (-0.10, 2.45) | .071 |
| BMI (kg/m^2^) | Run-in vs. baseline | -0.09 (-0.20, 0.03) | .151 | 0.01 (-0.17, 0.19) | .907 | -0.13 (-0.30, 0.05) | .159 |
|  | Post-int. vs. baseline | **-0.79 (-0.91, -0.67)** | **<.001** | **-0.96 (-1.14, -0.77)** | **<.001** | **-1.03 (-1.20, -0.85)** | **<.001** |
|  | 3-month FU vs. baseline | **-0.72 (-0.84, -0.60)** | **<.001** | **-0.96 (-1.15, 0.77)** | **<.001** | **-1.06 (-1.24, -0.88)** | **<.001** |
| Waist | Run-in vs. baseline | 0.02 (-0.60, 0.65) | .938 | 0.34 (-0.65, 1.33) | .501 | 0.56 (-0.37, 1.48) | .240 |
|  | Post-int. vs. baseline | **-2.98 (-3.61, -2.35)** | **<.001** | **-3.58 (-4.57, -2.59)** | **<.001** | **-3.53 (-4.46, -2.60)** | **<.001** |
|  | 3-month FU vs. baseline | **-2.15 (-2.80, 1.50)** | **<.001** | **-3.00 (-4.01, -1.99)** | **<.001** | **-3.02 (-3.98, -2.06)** | **<.001** |

**Supplementary Table 5. Intervention effects per compliance group**

|  |  | **Attendance (n=355)** | | **Attendance and MD post int. (n=159)** | | **Attendance and ΔMD (n=157)** | |
| --- | --- | --- | --- | --- | --- | --- | --- |
| **Outcomes** |  | **β (95%CI)** | ***p*** | **β (95%CI)** | ***p*** | **β (95%CI)** | ***p*** |
| Stool | Run-in vs. baseline | -0.03 (-0.32, 0.25) | .810 | -0.22 (-0.65, 0.22) | .324 | -0.09 (-0.51, 0.33) | .673 |
|  | Post-int. vs. baseline | **0.38 (0.10, 0.66)** | **.008** | 0.35 (-0.08, 0.79) | .111 | 0.26 (-0.16, 0.69) | .222 |
|  | 3-month FU vs. baseline | **0.42 (0.13, 0.71)** | **.005** | **0.55 (0.10, 0.99)** | **.016** | **0.46 (0.03, 0.90)** | **.037** |
| HADS anxiety | Run-in vs. baseline | 0.04 (-0.01, 0.09) | .096 | 0.02 (-0.06, 0.10) | .611 | 0.05 (-0.02, 0.13) | .161 |
|  | Post-int. vs. baseline | **-0.06 (-0.11, -0.01)** | **.013** | -0.08 (-0.16, -0.003) | .043^a^ | -0.07 (-0.15, 0.004) | .063 |
|  | 3-month FU vs. baseline | -0.03 (-0.08, 0.20) | .238 | 0.002 (-0.08, 0.08) | .952 | -0.01 (-0.09, 0.07) | .801 |
| HADS depression | Run-in vs. baseline | -0.02 (-0.08, 0.04) | .494 | -0.004 (-0.09, 0.08) | .936 | 0.03 (-0.06, 0.11) | .515 |
|  | Post-int. vs. baseline | **-0.14 (-0.19, -0.08)** | **<.001** | **-0.16 (-0.25, -0.07)** | **<.001** | **-0.19 (-0.28, -0.11)** | **<.001** |
|  | 3-month FU vs. baseline | **-0.13 (-0.19, -0.07)** | **<.001** | **-0.14 (-0.23, -0.05)** | **.003** | **-0.18 (-0.26, -0.09)** | **<.001** |
| CIS-20 subjective tiredness | Run-in vs. baseline | 0.82 (-0.13, 1.77) | .092 | 0.58 (-0.95, 2.12) | .457 | 1.33 (-0.20, 2.87) | .089 |
|  | Post-int. vs. baseline | **-3.00 (-3.96, -2.05)** | **<.001** | **-4.20 (-5.74, -2.66)** | **<.001** | **-4.24 (-5.77, -2.70)** | **<.001** |
|  | 3-month FU vs. baseline | **-2.02 (-3.01, -1.04)** | **<.001** | **-2.53 (-4.12, -0.94)** | **.002** | **-2.86 (-4.45, -1.28)** | **<.001** |
| MSNQ-p total | Run-in vs. baseline | 0.03 (-0.56, 0.63) | .911 | -0.39 (-1.29, 0.51) | .395 | 0.25 (-0.64, 1.14) | .586 |
|  | Post-int. vs. baseline | -0.33 (-0.92, 0.27) | .284 | -0.87 (-1.77, 0.03) | .057 | -0.39 (-1.29, 0.50) | .386 |
|  | 3-month FU vs. baseline | -0.37 (-0.98, 0.25) | .240 | **-1.10 (-2.03, -0.17)** | **.021** | -0.63 (-1.56, 0.29) | .179 |
| Nutrition \| Dietary compliance score | Run-in vs. baseline | -0.05 (-0.29, 0.19) | .680 | -0.18 (-0.52, 0.16) | .309 | **0.31 (0.01, 0.62)** | **.044** |
|  | Post-int. vs. baseline | **4.07 (3.83, 4.31)** | **<.001** | **5.45 (5.11, 5.79)** | **<.001** | **6.37 (6.07, 6.67)** | **<.001** |
|  | 3-month FU vs. baseline | **3.07 (2.82, 3.32)** | **<.001** | **3.96 (3.61, 4.31)** | **<.001** | **4.73 (4.42, 5.05)** | **<.001** |
| Exercise \| Moderate-vigorous | Run-in vs. baseline | 0.24 (-0.17, 0.65) | .253 | -0.18 (-0.78, 0.41) | .546 | 0.19 (-0.41, 0.78) | .543 |
|  | Post-int. vs. baseline | -0.13 (-0.54, 0.28) | .532 | 0.05 (-0.55, 0.64) | .880 | 0.19 (-0.41, 0.78) | .543 |
|  | 3-month FU vs. baseline | 0.32 (-0.10, 0.74) | .138 | 0.52 (-0.10, 1.14) | .102 | 0.72 (0.10, 1.35) | .024^a^ |

**Supplementary table 5 (continued)**

**Supplementary table 5 (continued)**

|  |  | **Attendance (n=355)** | | **Attendance and MD post int. (n=159)** | | **Attendance and ΔMD (n=157)** | |
| --- | --- | --- | --- | --- | --- | --- | --- |
| **Outcomes** |  | **β (95%CI)** | ***p*** | **β (95%CI)** | ***p*** | **β (95%CI)** | ***p*** |
| Exercise \| Strengthening | Run-in vs. baseline | -0.24 (-0.93, 0.44) | .486 | -0.35 (-1.51, 0.81) | .558 | -1.21 (-2.28, -0.14) | .026^a^ |
|  | Post-int. vs. baseline | -2.25e-17 (-0.69, 0.69) | 1.000 | 0.56 (-0.65, 1.78) | .362 | 0.33 (-0.80, 1.47) | .566 |
|  | 3-month FU vs. baseline | -0.59 (-1.28, 0.11) | .100 | -0.78 (-1.99, 0.42) | .202 | -0.47 (-1.59, 0.65) | .410 |
| Exercise \| Total | Run-in vs. baseline | 0.09 (-0.32, 0.50) | .673 | -0.25 (-0.86, 0.37) | .434 | 1.14e-17 (-0.62, 0.62) | 1.000 |
|  | Post-int. vs. baseline | -0.07 (-0.48, 0.35) | .751 | 0.10 (-0.52, 0.71) | .754 | 0.20 (-0.42, 0.81) | .532 |
|  | 3-month FU vs. baseline | 0.26 ( -0.16, 0.69) | .228 | 0.30 (-0.34, 0.93) | .357 | 0.68 (0.04, 1.32) | .038^a^ |
| Stress \| PSS total | Run-in vs. baseline | **0.70 (0.04, 1.36)** | **.038** | 0.13 (-0.94, 1.21) | .809 | 0.52 (-0.50, 1.53) | .320 |
|  | Post-int. vs. baseline | **-1.45 (-2.11, -0.79)** | **<.001** | **-2.15 (-3.22, -1.08)** | **<.001** | **-2.08 (-3.10, -1.07)** | **<.001** |
|  | 3-month FU vs. baseline | **-0.72 (-1.41, -0.04)** | **.038** | -0.97 (-2.08, 0.13) | .085 | **-1.18 (-2.23, -0.13)** | **.028** |
| Sleep \| MOS-SS Sleep disturbance | Run-in vs. baseline | -1.48 (-3.15, 0.19) | .082 | -1.53 (-4.18, 1.11) | .256 | -0.88 (-3.45, 1.70) | .505 |
|  | Post-int. vs. baseline | **-6.86 (-8.53, -5.19)** | **<.001** | **-7.86 (-10.51, -5.22)** | **<.001** | **-8.50 (-11.07, -5.92)** | **<.001** |
|  | 3-month FU vs. baseline | **-5.79 (-7.51, -4.06)** | **<.001** | **-7.21 (-9.94, -4.48)** | **<.001** | **-6.75 (-9.42, -4.08)** | **<.001** |
| Sleep \| MOS-SS Snoring | Run-in vs. baseline | -0.24 (-0.75, 0.27) | .361 | No convergence |  | No convergence |  |
|  | Post-int. vs. baseline | **-0.70 (-1.22, -0.18)** | **.008** | No convergence |  | No convergence |  |
|  | 3-month FU vs. baseline | -0.08 (-0.60, 0.45) | .781 | No convergence |  | No convergence |  |
| Sleep \| MOS-SS SOB headache | Run-in vs. baseline | -0.39 (-0.80, 0.02) | .063 | -0.25 (-0.87, 0.37) | .432 | -0.47 (-1.11, 0.17) | .149 |
|  | Post-int. vs. baseline | **-0.61 (-1.03, -0.20)** | **.004** | -0.25 (-0.87, 0.37) | .432 | -0.42 (-1.05, 0.22) | .199 |
|  | 3-month FU vs. baseline | -0.23 (-0.65, 0.19) | .281 | 0.08 (-0.55, 0.71) | .809 | -0.19 (-0.84, 0.46) | .564 |
| Sleep \| MOS-SS Sleep adequacy | Run-in vs. baseline | 0.93 (-1.40, 3.26) | .435 | 1.26 (-2.29, 4.80) | .487 | -0.64 (-3.96, 2.69) | .707 |
|  | Post-int. vs. baseline | **6.30 (3.97, 8.64)** | **<.001** | **8.24 (4.70, 11.78)** | **<.001** | **7.77 (4.45, 11.09)** | **<.001** |
|  | 3-month FU vs. baseline | **3.55 (1.14, 5.96)** | **.004** | 4.61 (0.95, 8.27) | .014^a^ | 4.37 (0.93, 7.81) | .013^a^ |

**Supplementary table 5 (continued)**

|  |  | **Attendance (n=355)** | | **Attendance and MD post int. (n=159)** | | **Attendance and ΔMD (n=157)** | |
| --- | --- | --- | --- | --- | --- | --- | --- |
| **Outcomes** |  | **β (95%CI)** | ***p*** | **β (95%CI)** | ***p*** | **β (95%CI)** | ***p*** |
| Sleep \| MOS-SS Sleep somnolence | Run-in vs. baseline | 1.16 (-0.41, 2.74) | .147 | 0.92 (-1.43, 3.28) | .443 | 0.89 (-1.43, 3.21) | .451 |
|  | Post-int. vs. baseline | **-5.84 (-7.42, -4.26)** | **<.001** | -7.55 (-9.90, -5.19) | **<.001** | **-7.56 (-9.88, -5.24)** | **<.001** |
|  | 3-month FU vs. baseline | **-3.42 (-5.05, -1.79)** | **<.001** | -4.61 (-7.04, -2.18) | **<.001** | **-5.06 (-7.47, -2.65)** | **<.001** |
| Sleep \| MOS-SS Sleep index 1 | Run-in vs. baseline | -0.37 (-1.72, 0.98) | .595 | -0.96 (-3.10, 1.18) | .377 | 0.17 (-1.86, 2.20) | .870 |
|  | Post-int. vs. baseline | **-5.18 (-6.54, -3.83)** | **<.001** | -6.18 (-8.32, -4.04) | **<.001** | **-6.26 (-8.30, -4.23)** | **<.001** |
|  | 3-month FU vs. baseline | **-3.71 (-5.11, -2.31)** | **<.001** | -4.74 (-6.95, -2.53) | **<.001** | **-4.56 (-6.66, -2.45)** | **<.001** |
| Sleep \| MOS-SS Sleep index 2 | Run-in vs. baseline | -0.87 (-2.15, 0.41) | .183 | -1.20 (-3.26, 0.86) | .254 | -0.30 (-2.29, 1.69) | .764 |
|  | Post-int. vs. baseline | **-5.92 (-7.20, -4.63)** | **<.001** | -7.17 (-9.23, -5.11) | **<.001** | **-7.23 (-9.22, -5.24)** | **<.001** |
|  | 3-month FU vs. baseline | **-4.14 (-5.46, -2.81)** | **<.001** | -5.13 (-7.25, -3.00) | **<.001** | **-4.96 (-7.02, -2.90)** | **<.001** |
| Sleep \| MOS-SS Sleep quantity | Run-in vs. baseline | 0.04 (-0.05, 0.13) | .390 | 0.09 (-0.04, 0.23) | .155 | 0.08 (-0.05, 0.22) | .241 |
|  | Post-int. vs. baseline | 0.08 (-0.01,0.17) | .093 | 0.11 (-0.02, 0.24) | .105 | **0.23 (0.09, 0.36)** | **.001** |
|  | 3-month FU vs. baseline | 0.05 (-0.04, 0.14) | .292 | 0.14 (0.003, 0.27) | .045^a^ | 0.09 (-0.05, 0.23) | .228 |
| Sleep \| MOS-SS Optimal sleep | Run-in vs. baseline | 0.23 (-0.19, 0.66) | .282 | -0.11 (-0.74, 0.53) | .745 | 0.49 (-0.16, 1.14) | .140 |
|  | Post-int. vs. baseline | 0.44 (0.01, 0.86) | .046^a^ | 0.16 (-0.48, 0.81) | .623 | 0.72 (0.06, 1.39) | .033^a^ |
|  | 3-month FU vs. baseline | **0.69 (0.24, 1.14)** | **.002** | 0.37 (-0.31, 1.04) | .285 | 0.69 (0.01, 1.38) | .048^a^ |

Baseline was coded as the reference; positive values therefore represent a *decrease* and negative value an *increase* from run-in to baseline. **Bold**=significant after multiple comparison correction (p-value was corrected for the number of scales analyzed per measures). ^a^ p<0.05; but not significant after multiple comparison correction. Abbreviations: MSIS-29 = Multiple Sclerosis Impact Score; SF-12 = Short Form health survey; BMI = Body Mass Index; HADS = Hospital Anxiety and Depression Scale; CIS-20 = Checklist Individual Strength-20-r; MSNQ-p = Multiple Sclerosis Neuropsychological Screening Questionnaire – patient version; Exercise = adherence to the Dutch exercise guidelines; MOS-SS = Medical Outcomes Study Sleep Scale; SOB = shortness of breath; PSS = Perceived Stress Scale; Post int. = Post-intervention; 3-mo. FU = 3-month follow-up measurement.

|  |  | **Normal weight (*n*=288)** | | **Overweight (*n*=174)** | | **Obese (*n*=104)** | |
| --- | --- | --- | --- | --- | --- | --- | --- |
| **Outcomes** |  | **β (95%CI)** | ***p*** | **β (95%CI)** | ***p*** | **β (95%CI)** | ***p*** |
| MSIS-29 physical | Run-in vs. baseline | 0.49 (-0.75, 1.73) | .436 | **1.90 (0.27, 3.54)** | **.022** | 0.43 (-1.65, 2.52) | .684 |
|  | Post-int. vs. baseline | **-2.41 (-3.67, -1.15)** | **<.001** | -1.34 (-3.00, 0.31) | .112 | **-5.29 (-7.38, -3.20)** | **<.001** |
|  | 3-month FU vs. baseline | -1.39 (-2.68, -0.10) | .034^a^ | -1.92 (-3.62, -0.22) | .027^a^ | **-4.32 (-6.51, -2.13)** | **<.001** |
| MSIS-29 psychological | Run-in vs. baseline | 0.58 (-0.97, 2.14) | .462 | **3.80 (1.90, 5.71)** | **<.001** | 1.90 (-0.56, 4.36) | .131 |
|  | Post-int. vs. baseline | **-3.16 (-4.74, -1.58)** | **<.001** | **-2.64 (-4.57, -0.71)** | **.007** | **-5.90 (-8.37, -3.44)** | **<.001** |
|  | 3-month FU vs. baseline | **-2.50 (-4.12, -0.89)** | **.002** | -1.99 (-3.98, -0.003) | .050 | **-3.44 (-6.02, -0.86)** | **.009** |
| SF12 physical health | Run-in vs. baseline | -0.70 (-1.41, 0.003) | .051 | -1.02 (-1.98, -0.06) | .036^a^ | -0.89 (-2.11, 0.34) | .156 |
|  | Post-int. vs. baseline | -0.08 (-0.80, 0.63) | .817 | 0.02 (-0.96, 0.99) | .976 | 0.92 (-0.31, 2.14) | .143 |
|  | 3-month FU vs. baseline | 0.18 (-0.55, 0.91) | .630 | 0.44 (-0.56, 1.45) | .387 | 1.30 (0.02, 2.57) | .047^a^ |
| SF12 mental health | Run-in vs. baseline | 0.62 (-0.36, 1.61) | .216 | -0.67 (-1.89, 0.56) | .286 | 0.34 (-1.14, 1.82) | .649 |
|  | Post-int. vs. baseline | **1.87 (0.88, 2.87)** | **<.001** | 0.93 (-0.31, 2.17) | .143 | **2.92 (1.44, 4.40)** | **<.001** |
|  | 3-month FU vs. baseline | 0.83 (-0.19, 1.85) | .111 | -0.07 (-1.35, 1.22) | .921 | 1.03 (-0.51, 2.58) | .190 |
| BMI (kg/m^2^) | Run-in vs. baseline | -0.08 (-0.30, 0.14) | .484 | -0.08 (-0.26, 0.11) | .430 | -0.21 (-0.48, 0.07) | .137 |
|  | Post-int. vs. baseline | **-0.39 (-0.61, -0.17)** | **.001** | **-0.96 (-1.15, -0.77)** | **<.001** | **-1.27 (-1.55, -0.99)** | **<.001** |
|  | 3-month FU vs. baseline | **-0.43 (-0.66, -0.20)** | **<.001** | **-0.91 (-1.10, -0.71)** | **<.001** | **-1.07 (-1.35, -0.78)** | **<.001** |
| Waist | Run-in vs. baseline | -0.12 (0.84, -0.59) | .735 | -0.49 (-1.45, 0.47) | .321 | 0.33 (-0.92, 1.57) | .608 |
|  | Post-int. vs. baseline | **-2.47 (-3.20, -1.74)** | **<.001** | **-3.41 (-4.38, -2.43)** | **<.001** | **-4.40 (-5.66, -3.14)** | **<.001** |
|  | 3-month FU vs. baseline | **-1.45 (-2.20, -0.70)** | **<.001** | **-2.42 (-3.43, -1.41)** | **<.001** | **-3.09 (-4.39, -1.78)** | **<.001** |

**Supplementary Table 6.** Intervention effects per BMI group

|  |  | **Normal weight (*n*=288)** | | **Overweight (*n*=174)** | | **Obese (*n*=104)** | |
| --- | --- | --- | --- | --- | --- | --- | --- |
| **Outcomes** |  | **β (95%CI)** | ***p*** | **β (95%CI)** | ***p*** | **β (95%CI)** | ***p*** |
| Stool | Run-in vs. baseline | **-0.34 (-0.65, -0.03)** | .**030** | 0.31 (-0.10, 0.72) | .136 | 0.07 (-0.46, 0.59) | .801 |
|  | Post-int. vs. baseline | **0.33 (0.02, 0.65)** | **.038** | **0.43 (0.02, 0.84)** | **.038** | 0.12 (-0.42, 0.65) | .670 |
|  | 3-month FU vs. baseline | 0.27 (-0.04, 0.59) | .091 | 0.41 (-0.01, 0.84) | .053 | 0.47 (-0.09, 1.04) | .099 |
| HADS anxiety | Run-in vs. baseline | **0.07 (0.02, 0.13)** | .**012** | **0.09 (0.02, 0.16)** | **.012** | 0.04 (-0.05, 0.12) | .403 |
|  | Post-int. vs. baseline | -0.04 (-0.10, 0.02) | .196 | -0.01 (-0.08, 0.07) | .878 | -0.09 (-0.18, -0.01) | .036^ |
|  | 3-month FU vs. baseline | -0.03 (-0.09, 0.03) | .392 | 0.001 (-0.07, 0.08) | .976 | -0.05 (-0.14, 0.04) | .250 |
| HADS depression | Run-in vs. baseline | -0.003 (-0.07, 0.06) | .920 | 0.02 (-0.06, 0.10) | .621 | -0.04 (-0.14, 0.07) | .473 |
|  | Post-int. vs. baseline | **-0.08 (-0.15, -0.02)** | .**011** | -0.06 (-0.14, 0.03) | .190 | **-0.20 (-0.30, -0.09)** | **<.001** |
|  | 3-month FU vs. baseline | -0.04 (-0.11, 0.02) | .202 | **-0.12 (-0.21, -0.03)** | **.006** | **-0.15 (-0.26, -0.04)** | **.008** |
| CIS-20 subjective tiredness | Run-in vs. baseline | 0.77 (-0.29, 1.84) | .155 | 1.21 (-0.11, 2.53) | .073 | 0.81 (-0.83, 2.45) | .334 |
|  | Post-int. vs. baseline | **-2.66 (-3.75, -1.58)** | **<.001** | **-2.30 (-3.65, -0.96)** | **.001** | **-4.10 (-5.75, -2.45)** | **<.001** |
|  | 3-month FU vs. baseline | **-1.82 (-2.93, -0.71)** | **.001** | -1.37 (-2.76, 0.01) | .052 | **-2.32 (-4.04, -0.60)** | **.008** |
| MSNQ-p total | Run-in vs. baseline | -0.05 (-0.73, 0.63) | .892 | 0.81 (-0.02, 1.64) | .055 | -0.20 (-1.29, 0.88) | .716 |
|  | Post-int. vs. baseline | -0.28 (-0.97, 0.42) | .432 | 0.33 (-0.51, 1.17) | .445 | **-1.27 (-2.36, -0.17)** | **.023** |
|  | 3-month FU vs. baseline | -0.14 (-0.85, 0.57) | .702 | -0.35 (-1.23, 0.52) | .430 | -0.69 (-1.83, 0.46) | .238 |
| Nutrition \| Dietary compliance score | Run-in vs. baseline | 0.14 (-0.13, 0.41) | .319 | -0.31 (-0.66, 0.04) | .081 | -0.15 (-0.62, 0.31) | .519 |
|  | Post-int. vs. baseline | **3.58 (3.30, 3.85)** | **<.001** | **3.64 (3.29, 4.00)** | **<.001** | **3.78 (3.31, 4.25)** | **<.001** |
|  | 3-month FU vs. baseline | **2.81 (2.53, 3.09)** | **<.001** | **2.54 (2.17, 2.90)** | **<.001** | **2.99 (2.50, 3.49)** | **<.001** |
| Exercise \| Moderate-vigorous | Run-in vs. baseline | 0.51 (0.04, 0.98) | .033^ | 0.34 (-0.23, 0.91) | .245 | -0.08 (-0.87, 0.71) | .840 |
|  | Post-int. vs. baseline | 0.05 (-0.42, 0.52) | .827 | -0.13 (-0.71, 0.45) | .649 | -0.49 (-1.29, 0.32) | .236 |
|  | 3-month FU vs. baseline | 0.41 (-0.07, 0.90) | .096 | 0.56 (-0.04, 1.17) | .068 | -0.37 (-1.22, 0.47) | .385 |

**Supplementary table 6 (continued)**

|  |  | **Normal weight (*n*=288)** | | **Overweight (*n*=174)** | | **Obese (*n*=104)** | |
| --- | --- | --- | --- | --- | --- | --- | --- |
| **Outcomes** |  | **β (95%CI)** | ***p*** | **β (95%CI)** | ***p*** | **β (95%CI)** | ***p*** |
| Exercise \| Strengthening | Run-in vs. baseline | -0.65 (-1.43, 0.13) | .104 | -0.55 (-1.48, 0.38) | .246 | 0.33 (-0.80, 1.46) | .567 |
|  | Post-int. vs. baseline | -0.46 (-1.25, 0.33) | .256 | -0.43 (-1.38, 0.52) | .374 | 1.03 (-0.20, 2.26) | .102 |
|  | 3-month FU vs. baseline | **-1.00 (-1.80, -0.20)** | **.014** | -0.58 (-1.56, 0.40) | .248 | 0.66 (-0.56, 1.89) | .289 |
| Exercise \| Total | Run-in vs. baseline | 0.18 (-0.27, 0.63) | .427 | 0.17 (-0.40, 0.74) | .562 | -1.08e-17 (-0.85, 0.85) | 1.000 |
|  | Post-int. vs. baseline | 0.02 (-0.44, 0.47) | .946 | -0.07 (-0.65, 0.50) | .800 | -0.08 (-0.93, 0.77) | .847 |
|  | 3-month FU vs. baseline | 0.21 (-0.26, 0.67) | .378 | 0.50 (-0.11, 1.10) | .106 | -0.09 (-0.98, 0.81) | .845 |
| Stress \| PSS total | Run-in vs. baseline | 0.37 (-0.40, 1.14) | .350 | 0.81 (-0.09, 1.71) | .077 | **1.63 (0.47, 2.79)** | **.006** |
|  | Post-int. vs. baseline | **-1.38 (-2.17, -0.59)** | **.001** | -0.82 (-1.74, 0.09) | .078 | -1.07 (-2.24, 0.10) | .073 |
|  | 3-month FU vs. baseline | -0.79 (-1.59, 0.02) | .057 | -0.66 (-1.61, 0.29) | .173 | -0.50 (-1.72, 0.72) | .422 |
| Sleep \| MOS-SS Sleep disturbance | Run-in vs. baseline | -1.09 (-2.92, 0.75) | .246 | -0.50 (-2.87, 1.86) | .676 | 0.05 (-3.03, 3.13) | .976 |
|  | Post-int. vs. baseline | **-4.33 (-6.20, -2.46)** | **<.001** | **-6.37 (-8.77, -3.96)** | **<.001** | **-8.49 (-11.60, -5.39)** | **<.001** |
|  | 3-month FU vs. baseline | **-3.87 (-5.79, -1.95)** | **<.001** | **-5.36 (-7.85, -2.87)** | **<.001** | **-4.88 (-8.11, -1.64)** | **.003** |
| Sleep \| MOS-SS Snoring | Run-in vs. baseline | -0.17 (-0.72, 0.37) | .531 | -0.31 (-1.01, 0.38) | .379 | No convergence |  |
|  | Post-int. vs. baseline | **-0.82 (-1.38, -0.26)** | .**004** | -0.32 (-1.03, 0.38) | .369 | No convergence |  |
|  | 3-month FU vs. baseline | 0.37 (-0.20, 0.94) | .206 | -0.66 (-1.40, 0.07) | .078 | No convergence |  |
| Sleep \| MOS-SS SOB headache | Run-in vs. baseline | -0.27 (-0.72, 0.18) | .234 | -0.17 (-0.75, 0.40) | .557 | -0.14 (-0.87, 0.59) | .710 |
|  | Post-int. vs. baseline | -0.30 (-0.76, 0.15) | .192 | -0.39 (-0.98, 0.20) | .198 | -0.67 (-1.42, 0.09) | .082 |
|  | 3-month FU vs. baseline | -0.33 (-0.79, 0.14) | .172 | -0.10 (-0.71, 0.50) | .740 | 0.53 (-0.24, 1.29) | .177 |
| Sleep \| MOS-SS Sleep adequacy | Run-in vs. baseline | 0.41 (-2.10, 2.92) | .750 | 0.17 (-3.03, 3.37) | .917 | -1.44 (-5.53, 2.65) | .489 |
|  | Post-int. vs. baseline | **4.80 (2.25, 7.36)** | **<.001** | **8.05 (4.80, 11.30)** | **<.001** | **5.92 (1.80, 10.03)** | **.005** |
|  | 3-month FU vs. baseline | 3.11 (0.49, 5.74) | .020 | 4.21 (0.84, 7.57) | .014^a^ | 1.77 (-2.52, 6.06) | .418 |

**Supplementary table 6 (continued)**

|  |  | **Normal weight (*n*=288)** | | **Overweight (*n*=174)** | | **Obese (*n*=104)** | |
| --- | --- | --- | --- | --- | --- | --- | --- |
| **Outcomes** |  | **β (95%CI)** | ***p*** | **β (95%CI)** | ***p*** | **β (95%CI)** | ***p*** |
| Sleep \| MOS-SS Sleep somnolence | Run-in vs. baseline | 0.73 (-1.00, 2.47) | .407 | 2.24 (-0.12, 4.59) | .063 | 0.64 (-2.07, 3.35) | .643 |
|  | Post-int. vs. baseline | **-5.47 (-7.24, -3.71)** | **<.001** | **-3.72 (-6.11, -1.33)** | **.002** | **-6.76 (-9.49, -4.03)** | **<.001** |
|  | 3-month FU vs. baseline | **-3.01 (-4.82, -1.19)** | **.001** | -2.21 (-4.69, 0.26) | .080 | **-4.77 (-7.61, -1.92)** | **.001** |
| Sleep \| MOS-SS Sleep index 1 | Run-in vs. baseline | -0.19 (-1.66, 1.28) | .803 | 0.08 (-1.79, 1.94) | .937 | 1.76 (-0.78, 4.31) | .175 |
|  | Post-int. vs. baseline | **-3.81 (-5.30, -2.31)** | **<.001** | **-5.30 (-7.20, -3.41)** | **<.001** | **-5.53 (-8.10, -2.97)** | **<.001** |
|  | 3-month FU vs. baseline | **-2.96 (-4.50, -1.42)** | **<.001** | **-4.04 (-6.00, -2.08)** | **<.001** | -2.01 (-4.68, 0.66) | .141 |
| Sleep \| MOS-SS Sleep index 2 | Run-in vs. baseline | -0.59 (-1.98, 0.81) | .411 | -0.12 (-1.91, 1.67) | .893 | 0.60 (-1.75, 2.94) | .617 |
|  | Post-int. vs. baseline | **-4.39 (-5.82, -2.97)** | **<.001** | **-5.67 (-7.49, -3.86)** | **<.001** | **-6.64 (-9.00, -4.28)** | **<.001** |
|  | 3-month FU vs. baseline | **-3.29 (-4.75, -1.83)** | **<.001** | **-3.94 (-5.82, -2.06)** | **<.001** | -3.07 (-5.53, -0.61) | .014^a^ |
| Sleep \| MOS-SS Sleep quantity | Run-in vs. baseline | -0.09 (-0.21, 0.04) | .161 | 0.04 (-0.10, 0.17) | .607 | 0.01 (-0.18, 0.19) | .940 |
|  | Post-int. vs. baseline | -0.03 (-0.15, 0.10) | .688 | 0.08 (-0.06, 0.22) | .273 | 0.21 (0.02, 0.40) | .026^a^ |
|  | 3-month FU vs. baseline | -0.07 (-0.20, 0.06) | .318 | 0.06 (-0.08, 0.20) | .415 | 0.05 (-0.14, 0.25) | .595 |
| Sleep \| MOS-SS Optimal sleep | Run-in vs. baseline | -0.27 (-0.74, 0.20) | .263 | **0.87 (0.23, 1.52)** | **.008** | 0.13 (-0.58, 0.85) | .716 |
|  | Post-int. vs. baseline | -0.13 (-0.61, 0.35) | .599 | **1.16 (0.50, 1.82)** | **.001** | 0.45 (-0.27, 1.18) | .223 |
|  | 3-month FU vs. baseline | 0.18 (-0.32, 0.68) | .483 | **0.90 (0.23, 1.58)** | **.009** | 0.29 (-0.46, 1.04) | .448 |

**Supplementary table 6 (continued)**

Baseline was coded as the reference; positive values therefore represent a *decrease* and negative value an *increase* from run-in to baseline. **Bold**=significant after multiple comparison correction (p-value was corrected for the number of scales analyzed per measures). ^a^ p<0.05; but not significant after multiple comparison correction. Abbreviations: MSIS-29 = Multiple Sclerosis Impact Score; SF-12 = Short Form health survey; BMI = Body Mass Index; HADS = Hospital Anxiety and Depression Scale; CIS-20 = Checklist Individual Strength-20-r; MSNQ-p = Multiple Sclerosis Neuropsychological Screening Questionnaire – patient version; Exercise = adherence to the Dutch exercise guidelines; MOS-SS = Medical Outcomes Study Sleep Scale; SOB = shortness of breath; PSS = Perceived Stress Scale; Post int. = Post-intervention; 3-mo. FU = 3-month follow-up measurement.

**Supplementary Table 7. Effect sizes for changes**

|  | Post-intervention vs. baseline | 3-month follow-up vs. baseline |
| --- | --- | --- |
| MSIS-29 physical | **0.32** | **0.25** |
| MSIS-29 psychological | **0.37** | **0.26** |
| SF-12 physical | 0.02 | 0.10 |
| SF-12 mental | **0.29** | 0.09 |
| BMI (Kg/m^2^) | **0.61** | **0.58** |
| Waist | **0.69** | **0.45** |
| HADS-anxiety | 0.11 | 0.07 |
| HADS-depression | **0.24** | **0.22** |
| CIS-20r Subjective fatigue | **0.43** | **0.27** |
| MSNQ-P | 0.07 | 0.07 |
| Nutrition \| dietary compliance score | **2.15** | **1.64** |
| Stress \| PSS | **0.25** | **0.15** |
| Sleep \| MOS-SS sleep disturbance | **0.50** | **0.40** |
| Sleep \| MOS-SS sleep adequacy | **0.39** | **0.21** |
| Sleep \| MOS-SS sleep somnolence | **0.48** | **0.29** |
| Sleep \| MOS-SS sleep index 1 | **0.51** | **0.34** |
| Sleep \| MOS-SS sleep index 2 | **0.37** | **0.25** |
| Sleep \| MOS-SS sleep quantity | 0.07 | 0.01 |

Effect sizes (Cohen’s d) derived by dividing the estimated coefficients from the linear mixed model by the residual standard deviation. **Bold=**medium effect size (0.14-0.55), **Bold=**large effect size (>0.55) according to Kinney et al., 2020 [ref: Kinney, A. R., Eakman, A. M., & Graham, J. E. (2020). Novel effect size interpretation guidelines and an evaluation of statistical power in rehabilitation research. *Archives of physical medicine and rehabilitation*, *101*(12), 2219-2226.] Abbreviations: MSIS-29 = Multiple Sclerosis Impact Score; SF-12 = Short Form health survey; BMI = Body Mass Index; HADS = Hospital Anxiety and Depression Scale; CIS-20 = Checklist Individual Strength-20-r; MSNQ-p = Multiple Sclerosis Neuropsychological Screening Questionnaire – patient version; MOS-SS = Medical Outcomes Study Sleep Scale; SOB = shortness of breath; PSS = Perceived Stress Scale;
